# Transcriptional Reprogramming of *Arabidopsis thaliana* Defence Pathways by the Entomopathogen *Beauveria bassiana* Correlates With Resistance Against a Fungal Pathogen but Not Against Insects

**DOI:** 10.3389/fmicb.2019.00615

**Published:** 2019-03-29

**Authors:** Maya Raad, Travis R. Glare, Helena L. Brochero, Caroline Müller, Michael Rostás

**Affiliations:** ^1^Bio-Protection Research Centre, Lincoln University, Lincoln, New Zealand; ^2^Facultad de Ciencias Agrarias, Universidad Nacional de Colombia, Bogotá, Colombia; ^3^Department of Chemical Ecology, Bielefeld University, Bielefeld, Germany; ^4^Department of Crop Sciences, Agricultural Entomology, University of Göttingen, Göttingen, Germany

**Keywords:** endophytes, glucosinolates, induced resistance, phytohormones, plant–microbe interaction, *Plutella xylostella*, *Myzus persicae*, *Sclerotinia sclerotiorum*

## Abstract

The entomopathogenic fungus *Beauveria bassiana* can adopt an endophytic lifestyle by colonising a wide array of plant species. *Beauveria*-colonised plants can show enhanced resistance against insects and plant pathogens alike. However, little is known about the molecular and physiological mechanisms that govern such interactions. Here, we assessed the effects of two *B. bassiana* strains (BG11, FRh2) on the growth of *Arabidopsis thaliana* and its resistance against two herbivore species and a phytopathogen. Plant responses were studied on the transcriptomic and metabolic level using microarrays and by measuring changes in defence-related phytohormones and glucosinolates (GLSs). Root inoculation with *B. bassiana* BG11 significantly increased plant growth, while FRh2 had no such effect. Both *Beauveria* strains decreased leaf lesion area caused by the phytopathogen *Sclerotinia sclerotiorum* but did not affect population growth of the aphid *Myzus persicae* or the growth of *Plutella xylostella* caterpillars. Microarray analyses of leaves from endophyte-inoculated *A. thaliana* provided evidence for transcriptional reprogramming of plant defence pathways, with strain-specific changes in the expression of genes related to pathogenesis, phytoalexin, jasmonic (JA), and salicylic acid (SA) signalling pathways. However, *B. bassiana* colonisation did not result in higher concentrations of JA and SA or major changes in leaf GLS profiles. We conclude that the endophyte *B. bassiana* induces plant defence responses and hypothesise that these contribute to enhanced resistance against *S. sclerotiorum*.

## Introduction

Entomopathogenic fungi are primarily known for their ability to penetrate the cuticle of insects and to cause disease. Once inside the host, the fungus proliferates and eventually grows out again through the cuticle to produce spores, which often persist in the soil until a new host can be infected (Hajek, 2004). Due to their insect-killing capabilities and the fact that some species are easily mass-produced, entomopathogenic fungi have been widely used as biopesticides (Glare et al., 2012). However, more recently an increasing number of studies have shown that entomopathogenic fungi can also adopt a lifestyle as endophytes by colonising plant tissues without causing disease symptoms (Schulz and Boyle, 2005; Roy et al., 2006; Card et al., 2016). This change in paradigm has opened new avenues of research into the ecology of insect-killing fungi and has raised interest in the question whether endophytic entomopathogens could be useful tools for sustainable crop protection (Lacey et al., 2015; Lugtenberg et al., 2016). Several studies have shown that symbioses between entomopathogens and plants can have positive impacts on plant growth and resistance (Vega et al., 2009; Vidal and Jaber, 2015; Jaber and Ownley, 2018; Vega, 2018).

The fungus *Beauveria bassiana* (Balsamo-Crivelli) Vuillemin was the first entomopathogen to be reported as a naturally occurring endophyte in maize (Bing and Lewis, 1991) and has since been isolated from several other plant species including coffee (Vega et al., 2010), white pine (Ganley and Newcombe, 2006) and common bean (Ramos et al., 2017). The fungus has also been successfully established as an endophyte in many crop species, with seed or root inoculation leading to systemic colonisation of aboveground plant parts (Vidal and Jaber, 2015; McKinnon et al., 2017). The potential benefits of endophytic *B. bassiana* on plant health such as improving plant growth and suppressing insect pests and plant pathogens have been demonstrated in several studies (McKinnon et al., 2017; Jaber and Ownley, 2018).

Despite accumulating reports on entomopathogens helping plants to fend off antagonists, very few authors have addressed the mechanisms underlying this phenomenon. Non-mutually exclusive hypotheses for explaining *B. bassiana*-mediated resistance against attacking organisms include the presence of fungal toxins and mycoparasitism (Vidal and Jaber, 2015; Sword et al., 2017; Jaber and Ownley, 2018). However, such mechanisms have either not been demonstrated or only for endophytic fungi other than *B. bassiana.* For example, toxic destruxins produced by the entomopathogen *Metarhizium robertsii* were detected in cowpea plants after root inoculation but whether concentrations would be high enough to affect herbivores remains unclear (Golo et al., 2014). Mycosis through direct infection of herbivores seems another plausible mechanism but has rarely been observed in plants containing endophytic entomopathogens (Vidal and Jaber, 2015). Finally, it has been suggested that fungus-induced plant responses could contribute to resistance against insects and pathogens (Ownley et al., 2008; Jaber and Ownley, 2018). Indeed, limited support for this notion is provided by a proteomic analysis of leaves of *Phoenix dactylifera* (Arecaceae), demonstrating that *B. bassiana* colonisation can lead to the induction of proteins related to stress and plant defences (Gomez-Vidal et al., 2009). So far, most of our knowledge on plant resistance mediated by beneficial endophytic fungi is derived from studies involving the root-colonising genus *Trichoderma*, which can parasitize other fungi but does not infect insects (Steyaert et al., 2003; Mukherjee et al., 2013; Pieterse et al., 2014; Vos et al., 2015).

Here, we assessed the effects of endophytic *B. bassiana* in promoting plant growth and resistance against three economically important pests and pathogens of Brassicaceae: the fungus *Sclerotinia sclerotiorum* (Ascomycota: Sclerotiniaceae) and the two herbivore species, *Myzus persicae* (Homoptera: Aphididae) and *Plutella xylostella* (Lepidoptera: Plutellidae). We conducted transcriptomic analyses of plant responses to two *B. bassiana* strains in the model plant *Arabidopsis thaliana* (Brassicaceae). This was complemented with measurements of defence hormones and glucosinolates (GLSs), the main secondary metabolites characteristic for members of the order Brassicales.

## Materials and Methods

### Plants and Entomopathogens

Surface sterilised and stratified *A. thaliana* seeds (ecotype Colombia Col-0) were grown on Murashige and Skoog basal medium (M5519 – Sigma-Aldrich, Castle Hill, NSW, Australia) in a growth chamber at 20–22°C, 60–70% RH under a 12L:12D day/night cycle. Two *B. bassiana* strains, FRh2 and BG11, were used in this study. *B. bassiana* strain BG11 was recovered from *Bellis perennis* (Asteraceae) (Bio-Protection Research Centre, Lincoln University), whereas *B. bassiana* strain FRh2 was recovered from *Hylastes ater* (Coleoptera: Curculionidae) (Reay et al., 2010).

### Endophyte Colonisation

For growth and plant resistance experiments, roots of 1-week-old *A. thaliana* seedlings, growing in a gnotobiotic environment, were dipped in a 10 ml of *B. bassiana* conidia suspension at a final concentration of 1 × 10^8^ conidia/ml for 1 min. Control plants were mock-inoculated with 0.05% Tween 80 in sterile water. Inoculated and control seedlings were grown in gamma-irradiated potting mix for 4 weeks in separate propagation boxes (Mini propagator, Hortlink New Zealand Ltd.).

For microarray, RT-qPCR, phytohormone and GLS analyses, roots of 5-week-old *A. thaliana* plants grown in a gnotobiotic environment were dipped in *B. bassiana* conidia suspension as described above. Inoculated and control plants were transplanted into twice autoclaved vermiculite and kept in separate autoclaved 4.2 l cereal containers for another 15 days.

All experiments consisted of three treatments: FRh2 inoculation (F), BG11 inoculation (B) and mock-inoculation (C) and were conducted using a completely randomised design in a growth chamber at 20–22°C, 60–70% RH under a 12L:12D day/night cycle expect for plant growth experiment where a cycle of 16L:8D day/night was used.

Colonisation of *A. thaliana* by each *B. bassiana* strain was assessed using standard isolation techniques on culture medium (Reay et al., 2010). Inoculated and mock-inoculated plants were divided into two parts, rosette and inflorescence. Plant parts were surface-sterilised for 1 min in 70% ethanol, 2 min in 4.2% NaOCl and then rinsed 3× 3 min in 0.01% Triton X-100 in sterile water. The sterilisation method was tested on aliquots of fungal conidia to confirm the effectiveness of the selected exposure time and NaOCl concentration on the viability of conidia. After sterilisation, imprints of each leaf and inflorescence were made on *Beauveria*-selective medium (BSM) to verify that plant surfaces were not contaminated (Brownbridge et al., 2012). Subsequently, the same leaf tissue and inflorescence were cut into segments of 0.5 to 1 cm, plated on BSM, and cultivated for up to 3 weeks at 20°C in a dark incubator. Emerging mycelia were isolated and identified on the basis of colony and conidia morphology. In addition, genomic DNA was extracted from *A. thaliana* rosettes and *B. bassiana* presence was further verified by PCR using SCAR primers developed by Castrillo et al. (2003). Three strain-specific molecular markers were tested to facilitate the detection of *B. bassiana* in field samples: SCA14_445_ (F 5′ TCTGTGCTGGCCCTTATCG 3′, R 5′ TCTGTGCTGGGTACTGACGTG 3′), SCA15_441_ (F 5′ TTCCGAACCCGGTTAAGAGAC 3′, R 5′ TTCCGAACCCATCATCCTGC 3′) and SCB9_677_ (F 5′ TGGGGGACTCGC AAA CAG 3′, R 5′ TGGGGGACTCAC TCC ACG 3′). Similar to Biswas et al. (2012), we found that SCA15_441_ amplified *B. bassiana* DNA best and was therefore used throughout this study. We tested 38 FRh2-inoculated, 34 BG11-inoculated and 51 control plants grown in gamma-irradiated soil for *B. bassiana* presence that had been used earlier in the bioassays. Additionally, plants grown in vermiculite under gnotobiotic conditions used for molecular (*n* = 4) and biochemical experiments (*n* = 6) were also tested for endophyte colonisation.

### Plant Growth

Plants were grown in gamma-irradiated soil and inoculated as described above. The following phenotypic traits were measured 4 weeks after inoculation: rosette diameter, number of leaves, shoot and root biomass (fresh weight) and root/shoot ratio. We also measured time until onset of the reproductive phase (appearance of inflorescence) and inflorescence length after 12 weeks. For each treatment *n* = 20 replicates were carried out.

### Testing of Plant Resistance Against Leaf-Chewing and Phloem-Sucking Herbivores

A colony of the diamondback moth *Plutella xylostella* was reared on potted cabbage plants (*Brassica oleracea* var. *capitata*) in a climate chamber at constant 22°C under a 16L: 8D day/night cycle. A single third-instar caterpillar was transferred to a 5-week-old *A. thaliana* plant inoculated with *B. bassiana* or a mock-inoculated control with 24–26 plants per treatment. Separate experiments were carried out for each endophyte strain with its corresponding control. Caterpillar body mass was measured before the experiment and after a feeding period of 48, 72, and 96 h.

*Myzus persicae* was maintained under the same conditions as described for *P. xylostella*. Five nymphs of *M. persicae*, characterised by their wide cauda, were carefully transferred to a 5-week-old *A. thaliana* plant inoculated with *B. bassiana* or a mock-inoculated control with 12–14 plants per treatment. Nymphs were caged onto a leaf by using a clip cage of 44 mm diameter. The cage was removed 24 h post infestation and only a single nymph, which had inserted its stylet, was left on the leaf to become reproductive. After 5 days the number of next generation nymphs was recorded daily for 10 days.

### Testing of Plant Resistance Against a Leaf Pathogen

Five-week-old *A. thaliana* plants inoculated with one of the *B. bassiana* strains and mock-inoculated controls were infected with *Sclerotinia sclerotiorum* using an agar plug method. The *S. sclerotiorum* strain SsOSR (Bio-Protection Research Centre, Lincoln University) was cultured on potato dextrose agar in a Petri dish of 9 cm diameter for 3 weeks at 20°C under a 12L:12D day/night cycle. A cork borer was used to punch out a 3 mm agar disc with fungal hyphae from a Petri dish. Discs were punched out 1 cm from the edge of the dish. The agar disc was placed on a single leaf surface. All selected leaves for infection were standardised for a length of ca. 7 cm. The agar discs were covered with plastic foil to maintain high relative humidity and plants were incubated in propagation boxes (Mini propagator, Hortlink New Zealand Ltd.). The experiment was carried out with 19–22 plants per treatment. Lesion areas were measured 5 days post infection according to the method of Rostás et al. (2006). The lesions were scanned and the area of each lesion was calculated using the self-written software Surface (available upon request from authors).

### Microarrays

Total RNA was extracted from inoculated and control *A. thaliana* rosettes of four independent biological replicates per treatment using the RNeasy Plant Mini Kit (QIAGEN). Each biological replicate consisted of a pool of eight plants. One-column DNase digestion treatment using the RNase-Free DNase Set (QIAGEN) was incorporated in the extraction protocol to eliminate any DNA contaminations in downstream experiments. RNA samples were sent to OakLabs (Hennigsdorf, Germany) for analysis using Agilent 8 × 60 K microarrays (Agilent Technologies, Santa Clara, CA, United States) with 32072 target IDs representing 30541 gene loci where annotation is based on *A. thaliana* Genome, TAIR10. RNA quality was confirmed by electrophoretic analysis via the 2100 Bioanalyzer (Agilent Technologies, Santa Clara, CA, United States) for determination of the RNA integrity number. The cRNA syntheses and microarray hybridisations were performed using the Low Input QuickAmp Labeling Kit and the Agilent Gene Expression Hybridisation Kit (Agilent Technologies). Microarray data were normalised according to the ranked median quantiles (Bolstad et al., 2003) using DirectArray software (OakLabs). Hierarchical clustering analysis (HCA) and principal component analysis (PCA) were used to test whether samples of the same treatment were homogenous and thus clustered together. DEGs were identified using two-sample *t*-tests with unequal variance (Welch’s *t*-test), with a *p*-value of 5% using DirectArray software. Data were further subjected to false discovery rate correction with a threshold of 5% (Benjamini and Hochberg, 1995). GO and enrichment analysis for each group of up- and downregulated DEGs were performed (Ashburner et al., 2000; Mi et al., 2017; The Gene Ontology Consortium, 2017). Gene expression data were visualised in the context of metabolic pathways using MapMan (Thimm et al., 2004; Usadel et al., 2009).

### Quantitative Real Time PCR

For microarray validation, the expression of seven genes was evaluated using RT-qPCR (Primer sequences: Supplementary Table S6). The selected genes were related to different defence responses and had shown differential regulation in the FRh2-inoculated plants transcriptome data. *AXR5* (Hardtke et al., 2007), *ASC4* (Abel et al., 1995), *MYB122* (Frerigmann and Gigolashvili, 2014) and *ARR11* (Kieber and Schaller, 2014) are involved in auxin, ET, camalexin and cytokinin pathways, respectively. *GLIP1* and chitinase gene are known to be involved in resistance against bacteria and fungi (Oh et al., 2005; Lee et al., 2009; Hermosa et al., 2012). *WRKY63* is involved in abiotic stress resistance and mediates plant responses to drought tolerance (Ren et al., 2010; Bakshi and Oelmuller, 2014).

Total RNA was extracted from FRh2-inoculated and mock-inoculated *Arabidopsis* rosette of three additional independent biological replicates using the RNeasy Plant Mini Kit and RNase-Free DNase Set (QIAGEN) as described above. RNA quality check was performed by electrophoresis and photometrical measurement with the Nanodrop 2000 spectrophotometer (Thermo Scientific). A total of 2 μg of DNase-treated RNA was then reverse-transcribed into the first-strand cDNA using SuperScript^®^ III First-Strand Synthesis System (Invitrogen). cDNA synthesis was performed according to the manufacturer’s instructions, using Oligo dB 12-18 primer and including an RNase H digestion as a last step to remove RNA template from the cDNA:RNA hybrid molecule. qPCR was performed in triplicate from three biological replicates with a reaction mixture containing gene specific primers, cDNA template with a dilution value of 1:10, SYBR Green reagent, ROX Reference Dye to normalise the fluorescent reporter signal and the FastStart^TM^ Taq DNA Polymerase, dNTPack (Roche). The thermal cycling conditions were 95°C for 10 min followed by 95°C for 15^′′^, 60°C for 45^′′^ and 72°C for 45^′′^ for 40 cycles, followed by melting curve step at 95°C for 15^′′^, 60°C for 1′ and 95°C for 15^′′^ to validate amplicon specificity. Non-template controls were included in each qPCR plate indicating the purity of the reagents. Primers were designed to amplify short cDNA fragments using Primer-BLAST^1^. The primers were designed to span an exon/exon junction with a product size between 70 and 100 bp using the RefSeq accession database. Three reference genes that were stable and not identified as differentially expressed in the FRh2-inoculated plants microarray data, actin 2 (*Act-2*); glyceraldehyde-3-phosphate dehydrogenase (*GAPDH*) and elongation factor (*EF1*α) genes were used as reference genes to normalise the qPCR data. The relative expression levels were analysed using the 2^-ΔΔCT^ method (Livak and Schmittgen, 2001) and are presented as log_2_ relative levels of gene expression.

### Phytohormone Analysis

Salicylic acid and jasmonic acid levels were quantified in *B. bassiana* inoculated and mock-inoculated *A. thaliana* leaves by vapour-phase extraction and subsequent gas chromatography-mass spectrometry (GC-MS) analysis according to Schmelz et al. (2004). Plants were grown and inoculated as described above in section Endophyte colonisation. The experiment was carried out with six independent replicates (plants) per treatment. Each replicate consisted of 150 mg of frozen homogenised leaf tissue. The plant material was extracted in micro-reaction tubes filled with zirconium beads (FastRNA^®^ Pro Green Kit), acidified 1-propanol in water and methylene chloride, using a FastPrep^®^-24 System (MP Biomedicals, Solon, OH, United States). For quantification, D6-salicylic acid (CDN Isotopes, Pointe-Claire, QC, Canada) and dihydrojasmonic acid (TCI America, United States) were used as internal standards. Trimethylsilyldiazomethane (Sigma-Aldrich, Castle Hill, NSW, Australia) was added to derivatise the two phytohormones into their corresponding methyl esters. Samples were subjected to a vapour-phase extraction procedure consisting of two evaporation steps at 70°C and 200°C using a volatile collection trap packed with 30 mg Super-Q absorbent (Analytical Research Systems, Micanopy, FL, United States). The absorbed methylated compounds were then eluted from the collection trap with methylene chloride and stored at -80°C for subsequent GC-MS analysis according to Maag et al. (2014).

### Glucosinolate Analysis

Glucosinolate concentrations were measured in inoculated and mock-inoculated *A. thaliana* leaves as described above. The experiment was carried out with six independent replicates (plants) per treatment. Freeze dried leaves samples of each replicate were pulverised in 2 ml microcentrifuge tubes containing 2.5 mm zirconium/silica beads (dnature, New Zealand) using a bead mill (TissueLyser II, Qiagene, Hilden, Germany) for 1 min. Leaf samples (10 mg) were then extracted three times in 80% methanol. At the first extraction, 2-propenyl glucosinolate (Phytoplan, Heidelberg, Germany) was added as internal standard. Purified sulfatase (E.C. 3.1.6.1; purification following; Graser et al., 2001) was used to convert glucosinolates to desulfoglucosinolates which were then analysed by high performance liquid chromatography (HPLC) coupled to a diode array detector (HPLC-1200 Series, Agilent Technologies, Inc., Santa Clara, CA, United States) as described by Abdalsamee and Müller (2012). Desulfoglucosinolate separation was performed on a reverse phase Supelcosil LC 18 column (3 μm, 150 × 3 mm, Supelco, Bellefonte, PA, United States) using a gradient of water to methanol, starting at 5% methanol for 6 min, and increasing from 5 to 95% methanol within 13 min with a hold at 95% for 2 min. Metabolites were identified by comparison of retention times and UV spectra to purified standards (Phytoplan, Heidelberg, Germany; Copenhagen, Denmark) or by confirming the identities by ultra-HPLC coupled with a time of flight mass spectrometer (1290 Infinity UHPLC and 6210 TOF-MS Agilent, Technologies, Santa Clara, CA, United States). Desulfoglucosinolates were quantified using the integrated area at 229 nm, applying the response factors as described previously (Brown et al., 2003), and relating the amounts to the sample dry mass.

### Statistical Analyses

Differences in presence/absence between *B. bassiana* strains in *A. thaliana* tissues was analysed by a Chi-square test. Plant phenotypic traits, concentrations of JA and several GLSs were analysed using one-way ANOVA followed by Fisher’s least significant difference (LSD) *post hoc* tests. Data not meeting assumptions of normality and homogeneity of variance, such as number of leaves, root/shoot ratio, SA concentration, 4-hydroxyindol-3-ylmethyl GLS and 5-methylsulfinylpentyl GLS levels were analysed using the non-parametric Kruskal–Wallis ANOVA followed by a Student–Newman–Keuls test. A PCA was performed to visualise the effects of *B. bassiana* strains on glucosinolate concentrations. The biomass of *P. xylostella* caterpillars and population growth of *M. persicae* aphids were analysed using Student’s *t*-tests for independent samples and repeated measures ANOVA, respectively. Differences in mortality between caterpillars reared on endophyte-inoculated and control plants were compared by Chi-square tests. Count data for the population growth of aphids of *M. persicae* were square root transformed and homogeneity of variance was tested using Cochran’s test. *S. sclerotiorum* assay was analysed using one-way ANOVA followed by Fisher’s least significant difference (LSD) *post hoc* tests. *S. sclerotiorum* lesions area data was log-transformed to meet assumption of normality and homogeneity of variance. Statistical analyses were performed using IBM^®^ SPSS statistics 22 and Statistica 13 software as well as R package version 3.2.0^2^.

## Results

### Endophytic Colonisation of *A. thaliana* by the Fungal Entomopathogen *B. bassiana*

The entomopathogenic fungus was able to endophytically colonise aboveground tissues of the model plant *A. thaliana*. The root dipping method showed that the fungus can translocate systemically throughout the plant system when roots were dipped in conidia suspension, confirming previous studies (e.g., Muvea et al., 2014; Qayyum et al., 2015).

No statistically significant differences in colonisation rates were found between the strains FRh2 and BG11 (χ^2^: 2.62, d.f. = 1, *P* = 0.106). However, the environmental conditions of the inoculated plants had a strong influence on endophyte presence. While 100% of inoculated plants grown in vermiculite and under germ-free conditions were infected with *B. bassiana*, only 53 ± 6% of plants were tested positive when grown in soil under non-gnotobiotic conditions. No background infection of *B. bassiana* in *A. thaliana* seeds and seedlings was found using both molecular detection and the standard isolation techniques on culture medium. The fungus was recovered from rosettes and inflorescences after root inoculation, suggesting systemic colonisation of the entire plant. None of the control plants were colonised by the fungus. Also, tissue imprints and cuts showed no presence of *B. bassiana*, indicating that the fungus did not colonise the plant surface. Using PCR, we were able to detect *B. bassiana* in inoculated but not in control plants.

### Growth Promotion Depends on Entomopathogen Strain

**Figure 1:**
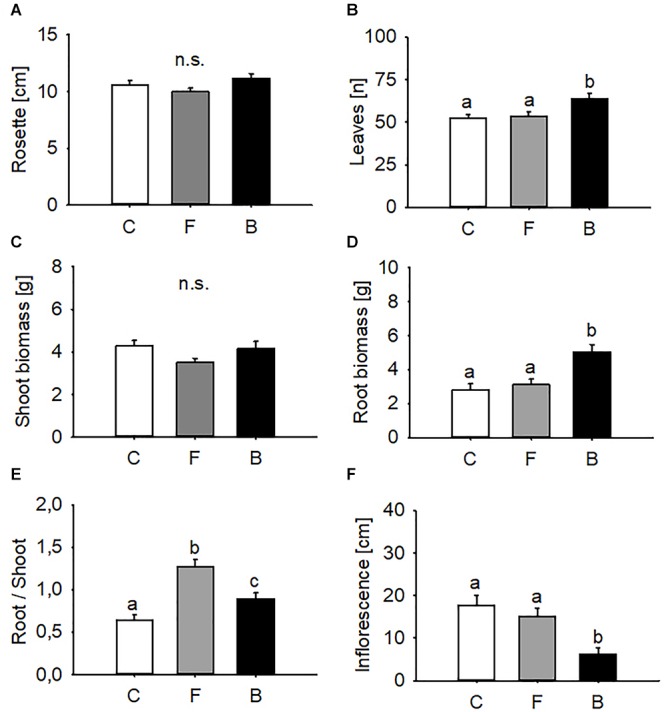
Effects of *B. bassiana* on *A. thaliana* growth. Rosette diameter **(A)**, numbers of leaves **(B)**, shoot biomass **(C)**, root biomass **(D)**, root/shoot ratio **(E)**, and inflorescence length **(F)** in *B. bassiana* colonised (F, FRh2 and B, BG11) and control (C) plants. Error bars represent the standard error of the mean (*N* = 20), bars with different letters differ significantly (*P* < 0.05).

Endophytic colonisation by *B. bassiana* showed strain-specific effects on several phenotypic traits of *A. thaliana.* Rosette diameter remained unaffected by the endophyte (one-way ANOVA, d.f. = 2, *F* = 2.336, *P* = 0.106; Figure 1A) but plants infected with BG11 produced significantly more leaves than either controls or FRh2-treated *A. thaliana* (Kruskal–Wallis ANOVA, *H* = 7.461, d.f. = 2, *P* = 0.024; BG11 vs. control: *P* = 0.037, BG11 vs. FRh2: *P* < 0.001, FRh2 vs. control: *P* = 0.761; Figure 1B). Likewise, no statistically significant effect was found for shoot biomass (one-way ANOVA, d.f. = 2, *F* = 2.519, *P* = 0.089; Figure 1C), while root biomass was increased in BG11 plants (one-way ANOVA, d.f. = 2, *F* = 10.727, *P* < 0.001; BG11 vs. control: *P* < 0.001, BG11 vs. FRh2: *P* = 0.001, FRh2 vs. control: *P* = 0.845; Figure 1D). Plants of all three treatments differed in their root/shoot ratios (Kruskal–Wallis ANOVA, d.f. = 2, *H* = 27.307, *P* < 0.001; BG11 vs. control: *P* = 0.002, BG11 vs. FRh2: *P* < 0.001, FRh2 vs. control: *P* < 0.001; Figure 1E). The time until plants entered the reproductive phase was independent of endophyte colonisation (Kruskal–Wallis ANOVA, d.f. = 2, *H* = 2.293, *P* = 0.318) and reached on average 29 ± 1.0 days (control), 29 ± 1.5 days (BG11) and 29 ± 0.3 days (FRh2). However, BG11 treated plants produced significantly shorter inflorescences (one-way ANOVA, d.f. = 2, *F* = 9.198, *P* < 0.001; BG11 vs. control: *P* < 0.001, BG11 vs. FRh2: *P* = 0.007, FRh2 vs. control: *P* = 0.624; Figure 1F).

### Entomopathogen Colonisation Increases Plant Resistance to Leaf Pathogen but Not to Insect Herbivores

**Figure 2:**
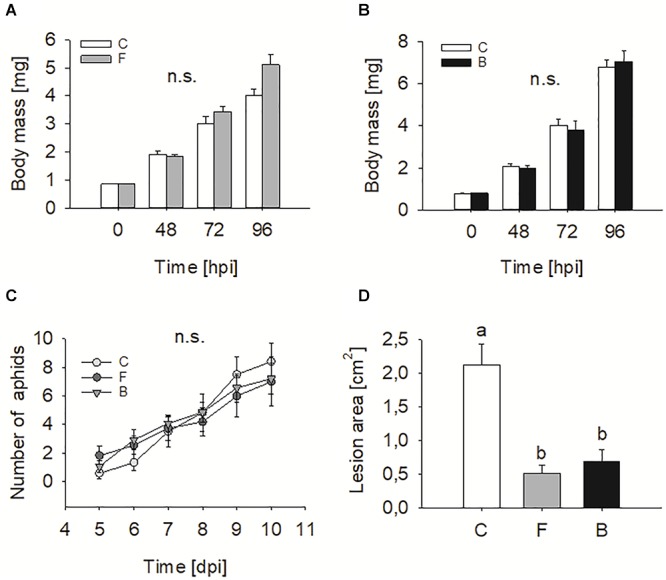
Effects of endophytic *B. bassiana* on two insect herbivores and a leaf pathogen. *P. xylostella* body mass (means ± SE) after 48, 72, and 96 h of feeding on *B. bassiana* FRh2 **(A)** and BG11 **(B)** colonised *Arabidopsis thaliana* (*N* = 24–26). *M. persicae* population growth when feeding on control and *B. bassiana* colonised *A. thaliana* **(C)**. Symbols represent mean number of *M. persicae* (*N* = 12–14). *S. sclerotiorum* lesion area on *A. thaliana* leaf measured 5 days post infection **(D)**. Disease intensity was calculated as average lesion area (*N* = 19–22). Different letters above bars indicate significant differences (*P* < 0.05). C, control plants; F, FRh2 inoculated plants; B, BG11 inoculated plants. Error bars represent the standard error of the mean. n.s., not significant.

Endophyte colonisation had no effect on the mortality rate of *P. xylostella* caterpillars, which was 31% and 38% in FRh2- and BG11-treated plants, respectively. Mortality in the corresponding controls was 29% in both experiments (Chi-square tests, FRh2 vs. control: χ^2^ = 0.02, d.f. = 1, *P* = 0.902; BG11 vs. control: χ^2^ = 0.38, d.f. = 1, *P* = 0.540). No significant differences in *P. xylostella* caterpillar body mass were observed after 48, 72, and 96 h of feeding on *B. bassiana*-inoculated plants when compared to insects feeding on control plants (*t*-test for FRh2 at 0 h; *t* = 0.39, *P* = 0.969, *n* = 24–26; 48 h; *t* = 0.502, *P* = 0.618, *n* = 22–24; 72 h; *t* = -1.228, *P* = 0.227, *n* = 20–19; 96 h; *t* = -1.959, *P* = 0.059, *n* = 17–18; *t*-test for BG11 at 0 h; *t* = -0.607, *P* = 0.547, *n* = 23–24; 48 h; *t* = 0.459, *P* = 0.649, *n* = 20–22; 72 h; *t* = 0.377, *P* = 0.709, *n* = 20; and 96 h; *t* = -0.345, *P* = 0.732, *n* = 15–17, Figure 2A,B).

Equally, the population growth of aphids of *M. persicae* was not significantly affected by *B. bassiana* presence when fixed on plants within a 10-day period (repeated measures ANOVA, treatment effect: d.f. = 2, *F* = 0.37, *P* = 0.692, Figure 2C).

In contrast to insect bioassays, inoculation with *B. bassiana* resulted in a significant reduction in *S. sclerotiorum* lesion area on leaves compared to those on mock-inoculated plants (one-way ANOVA, d.f. = 2, *F* = 22.062, *P* < 0.001, Figure 2D). The effect was significant for both *B. bassiana* strains compared to the control plants.

### Entomopathogen Colonisation Results in Strain-Specific Remodelling of the *A. thaliana* Transcriptome

**Figure 3:**
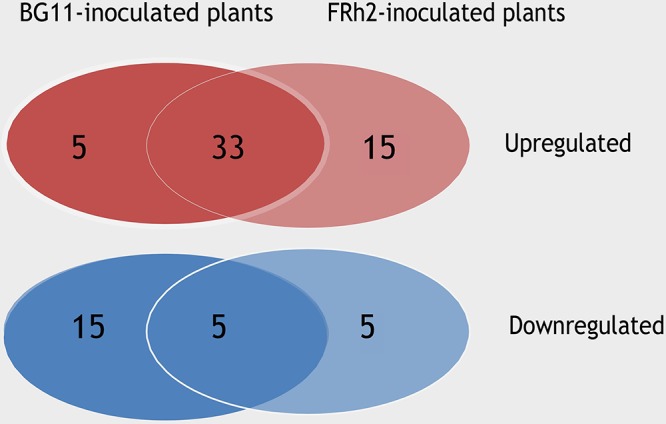
Venn diagram comparing the expression patterns of the shared differentially expressed genes (DEGs) between FRh2- and BG11-inoculated plants.

The comparative transcriptome analysis of *B. bassiana*-inoculated (FRh2, BG11) and mock-inoculated plants revealed 1,166 differentially expressed genes (DEGs) for FRh2-inoculated plants and 552 DEGs for BG11-inoculated plants, indicating a strain-specific effect on the plant’s gene expression. The ratio of upregulated to downregulated *A. thaliana* transcripts was 58:42% in FRh2-inoculated plants and 52:48% in BG11-inoculated plants (mean of four biological replicates). Fifty-eight DEGs were shared between the FRh2-inoculated plants and BG11-inoculated plants. Thirty-eight of the 58 shared DEGs had a similar expression pattern in the presence of FRh2 or BG11 while the remaining showed diverging expression patterns (Figure 3 and Supplementary Table S5).

Similarities between FRh2- and BG11-inoculated plants were observed in the upregulation of defence-related processes. Only BG11-inoculated plants showed responses associated to wounding (Table 1), however, both fungal strains induced genes involved in oxidative stress processes. FRh2-inoculated plants furthermore showed transcriptional changes in many additional abiotic stress-related processes that included responses to heat and temperature, while BG11-inoculated plants showed downregulation of genes related to radiation and light processes (Table 1 and Supplementary Tables S1–S4). An apparent difference in DEGs between FRh2- and BG11-inoculated plants was observed in the biological processes relating to plant hormones: DEGs associated with responses to JA, ethylene (ET) and SA were upregulated in BG11-inoculated plants, whereas DEGs associated with gibberellin and auxin stimulus were downregulated in FRh2-inoculated plants compared to control plants (Table 1 and Supplementary Tables S1–S4).

**Table 1:** Gene ontology terms and enrichment analysis for FRh2- and BG11-inoculated plants.

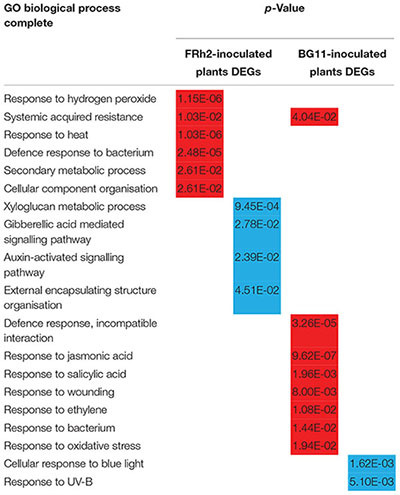

Red and blue boxes show up- and downregulated biological process enriched at *p*-value < 0.05.

**Figure 4:**
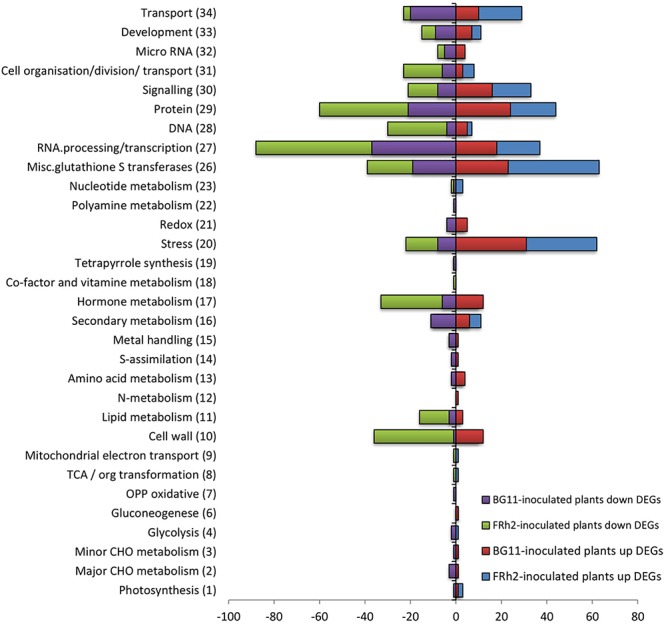
Numbers of differentially expressed genes (DEGs) assigned into MapMan bins (bin number in brackets) from FRh2- and BG11-inoculated plants. Downregulated DEGs are negative. Unassigned DEGs were 142, –225, and 61, –98 for FRh2- and BG11-inoculated plants, respectively. OPP, oxidative pentose phosphate; TCA/org transformation, tricarboxylic acid cycle/organic acid transformation.

Gene ontology (GO) and enrichment analysis were complemented by mapping the transcriptomic data with MapMan. An overview of the plant’s metabolic pathways that were affected by *B. bassiana* colonisation was generated. MapMan mapped 1,163 and 548 DEGs into the 35 major bins for FRh2- and BG11-inoculated tissues, respectively. Bins 7, 12, 14 (oxidative pentose phosphate, N-metabolism, S-assimilation, polyamine metabolism) and bins 8, 9, 18 (tricarboxylic acid cycle/organic acid transformation, mitochondrial electron transport/ATP synthesis, co-factor and vitamin metabolism) were the only non-represented bins for FRh2- and BG11-inoculated plants, respectively (Figure 4). A total of 367 DEGs (142 upregulated and 225 downregulated) for FRh2-inoculated plants and 159 DEGs (61 upregulated and 98 downregulated) for BG11-inoculated plants were unassigned (bin 35) DEGs (Figure 4).

**Figure 5:**
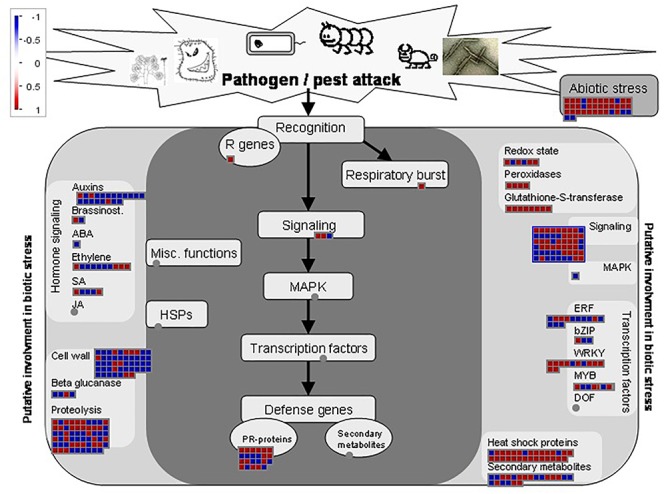
Biotic stress overview pathway for FRh2-inoculated plants DEGs as generated by MapMan software. Red and blue boxes represent up and downregulated genes, respectively. Colour intensity represents the degree of expression given as log_2_ fold change >1 and <–1.

**Figure 6:**
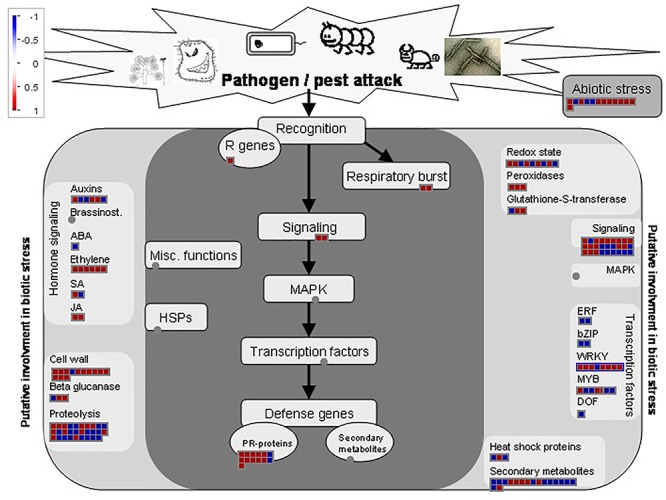
Biotic stress overview pathway for BG11-inoculated plants DEGs as generated by MapMan software. Red and blue boxes represent up and downregulated genes, respectively. Colour intensity represents the degree of expression given as log_2_ fold change >1 and <–1.

The biotic stress overview pathway generated by MapMan highlighted the involvement of both FRh2- and BG11-inoculated plants in defence-related responses and gave a visual assessment on how *B. bassiana* reprograms major parts of the plant immune response (Figure 5, 6). There were changes to expressions of stress-related transcripts associated to biotic and abiotic stress. MapMan identified 374 and 175 DEGs associated with the biotic stress overview pathway for FRh2- and BG11-inoculated plants, respectively. Among these, only 38 DEGs for FRh2-inoculated plants and 13 DEGs for BG11-inoculated plants were related to abiotic stress (bin 20.2) whereas the remaining were related directly or indirectly to biotic stress. The majority of the 38 FRh2-inoculated plants transcripts related to abiotic stress were identified as heat shock protein coding genes. A subset of 23 DEGs for FRh2-inoculated plants and 13 DEGs for BG11-inoculated plants were related directly to biotic stress (bin 20.1) and mapped as pathogenesis-related proteins coding genes (bin 20.1.7). The remaining of FRh2- and BG11-inoculated plants DEGs were indirectly related to biotic stress responses and mapped as signalling (bin 30), transcription factors (bin 27), oxidative stress (bin 21 and bin 26), hormones (bin 17), secondary metabolism-related genes (bin 16) and cell-wall modification genes (bin 10). For both FRh2- and BG11-inoculated plants DEGs, regulation of transcription showed diverging expression patterns in DEGs identified as WRKY factors, MYB domain and ET response factors AP2/ERF coding genes. Transcripts of FRh2- and BG11-inoculated plants associated with oxidative stress were identified as glutaredoxins, peroxidases and glutathione *S* transferases (GST) coding genes. DEGs associated with the secondary metabolism were related to camalexin and flavonoid metabolism and those associated with hormones metabolism were identified as auxin-responsive protein, ET signal transduction coding genes and JA and SA metabolism-associated genes.

The expression levels of the selected genes followed the same pattern of expression as in the FRh2-inoculated plants microarray. Averaging the expression level of each gene over the three biological replicates and over the three reference genes showed that *AXR5*, *ACS4*, and *ARR1* expression were downregulated, whereas *GLIP1*, chitinase gene, *MYB122* and *WRKY63* expression were upregulated (Supplementary Figure S1).

### Entomopathogen Colonisation Affects Secondary Metabolite Profiles but Not Defence Hormones

**Figure 7:**
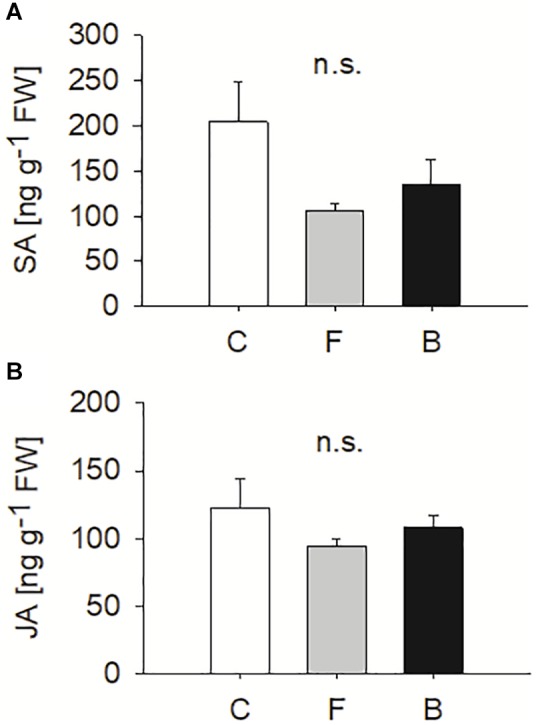
Levels of salicylic acid **(A)** and jasmonic acid **(B)** (means ± SE) measured in *B. bassiana* colonised (F, FRh2 and B, BG11) and control (C) *A. thaliana*. Error bars represent the standard error of the mean (*N* = 6), bars with different letters differ significantly (*P* < 0.05). n.s., not significant.

Colonisation with *B. bassiana* did not result in increased concentrations of JA and SA in *A. thaliana* leaves 15 days after inoculation. For the three treatments, no significant differences (*P* > 0.05) were found in SA and JA concentrations (one-way ANOVA for JA d.f. = 2, *F* = 0.908, *P* = 0.426, Figure 7; Kruskal–Wallis Test for SA, d.f. = 2, χ^2^ = 0.1.333, *P* = 0.513, Figure 7).

**Figure 8:**
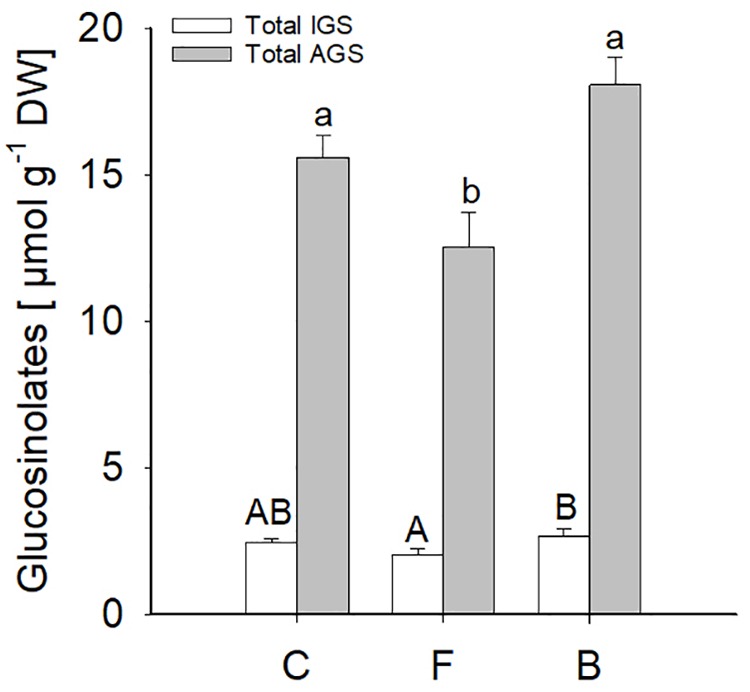
Indole (white bars) and aliphatic (grey bars) glucosinolate levels (means ± SE) measured in *B. bassiana* colonised (F, FRh2 and B, BG11) and control (C) *A. thaliana* leaves. Error bars represent the standard error of the mean (*N* = 6), bars with different letters differ significantly, (*P* < 0.05).

**Figure 9:**
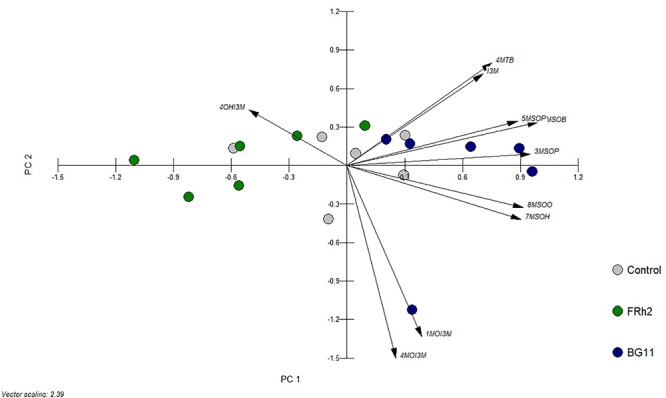
Bi-plot of principal component analysis (PCA) of glucosinolates in *A. thaliana* leaves. Plants were mock-inoculated (control) or colonised by *B. bassiana* strains FRh2 and BG11, respectively. Glucosinolates (GLS): 3MSOP, 3-methylsulfinylpropyl GLS; 4MSOB, 4-methylsulfinylbutyl GLS; 5MSOP, 5-methylsulfinylpentyl GLS; 6MSOH, 6-methylsulfinyl-heptyl GLS; 7MSOH, 7-methylsulfinylheptyl GLS; 4MTB, 4-methylthiobutyl GLS; 8MSOO, 8-methylsulfinyl-octyl GLS; 4MOI3M, 4-methoxyindol-3-ylmethyl GLS; 1MOI3M, 1-methoxyindol-3-ylmethyl GLS; 4OHI3M, 4-hydroxyindol-3-ylmethyl GLS; I3M, indol-3-ylmethyl GLS. PC1 = 49%, PC2 = 17%.

Slight changes in leaf GLS concentrations were observed due to entomopathogen colonisation after 15 days of inoculation. No significant differences (*P* ≤ 0.05) in total indole glucosinolate (IGS) levels (sum of four individual indole GLS: 4MOI3M, 4-methoxyindol-3-ylmethyl GLS; 4OHI3M, 4-hydroxyindol-3-ylmethyl GLS; I3M, indol-3-ylmethyl GLS; 1MOI3M, 1-methoxyindol-3-ylmethyl GLS) were observed between *B. bassiana*-treated and control plants. However, total aliphatic glucosinolate (AGS) levels (sum of seven individual aliphatic GLS: 3MSOP, 3-methylsulfinylpropyl GLS; 4MSOB, 4-methylsulfinylbutyl GLS; 5MSOP, 5-methylsulfinylpentyl GLS; 6MSOH, 6-methylsulfinyl-heptyl GLS; 7MSOH, 7-methylsulfinylheptyl GLS; 4MTB, 4-methylthiobutyl GLS; 8MSOO, 8-methylsulfinyl-octyl GLS) were affected by the fungus (one-way ANOVA, IGS: d.f. = 2, *F* = 2.886, *P* = 0.087; AGS: d.f. = 2, *F* = 7.958, *P* = 0.004, Figure 8). FRh2-colonised plants showed lower levels of AGS compared to controls with a significantly low level of 4MSOB (one-way ANOVA, d.f. = 2, *F* = 6.676, *P* = 0.008, Table 2). Despite the absence of a significant change in the total amount of AGS when compared to controls, BG11-colonised plants showed a significant increase in the level of 7MSOH, 4MTB, 8MSOO) when compared to FRh2-colonised plants (one-way ANOVA, d.f. = 2, 7MSOH: *F* = 14.146, *P* < 0.001; 4MTB: *F* = 7.843, *P* = 0.005, 8MSOO: *F* = 8.619, *P* = 0.003, Table 2). PCA resulted in distinct clusters of FRh2- and BG11-treated plants, which overlapped with the cluster of mock-inoculated controls (Figure 9).

**Table 2:** Mean (±SE) concentration of glucosinolates (GLS) (μmol g^-1^ dry weight) in *Arabidopsis thaliana* leaves of *Beauveria bassiana*-colonised plants (F, FRh2; B, BG11; C, control).

Glucosinolates^a^	*C*	*F*	*B*
Total AG	15.60 ± 0.75	12.54 ± 1.18*	18.07 ± 0.95
Total IG	2.44 ± 0.11	2.01 ± 0.21	2.67 ± 0.23
3MSOP	1.27 ± 0.05	0.99 ± 0.12	1.47 ± 0.12
4MSOB	12.32 ± 0.56	9.91 ± 0.91*	13.79 ± 0.76
5MSOP	1.07 ± 0.03	0.90 ± 0.10	1.17 ± 0.06
6MSOH	0.08 ± 0.02	0.06 ± 0.01	0.09 ± 0.01
7MSOH	0.19 ± 0.03	0.13 ± 0.02	0.30 ± 0.02*
4MTB	0.15 ± 0.04	0.11 ± 0.02	0.38 ± 0.08*
8MSOO	0.52 ± 0.09	0.43 ± 0.08	0.88 ± 0.07*
4MOI3M	0.47 ± 0.04	0.48 ± 0.06	0.62 ± 0.08
1MOI3M	0.29 ± 0.08	0.15 ± 0.03	0.31 ± 0.09
4OHI3M	0.02 ± 0.01	0.04 ± 0.01	0.00 ± 0.00
I3M	1.66 ± 0.10	1.34 ± 0.20	1.74 ± 0.021

*Asterisk indicates statistically significant difference compared to control plant. One-way ANOVA followed by Fisher’s least significant difference (*P* ≤ 0.05). ^*a*^AG, aliphatic glucosinolates; IG, indole glucosinolates; 3MSOP, 3-methylsulfinylpropyl GLS; 4MSOB, 4-methylsulfinylbutyl GLS; 5MSOP, 5-methylsulfinylpentyl GLS; 6MSOH, 6-methylsulfinyl-heptyl GLS; 7MSOH, 7-methylsulfinylheptyl GLS; 4MTB, 4-methylthiobutyl-GLS; 8MSOO, 8-methylsulfinyl-octyl GLS; 4MOI3M, 4-methoxyindol-3-ylmethyl GLS; 1MOI3M, 1-methoxyindol-3-ylmethyl GLS; 4OHI3M, 4-hydroxyindol-3-ylmethyl GLS; I3M, indol-3-ylmethyl GLS*.

## Discussion

In this study, we assessed the ability of *B. bassiana* to promote plant growth and we tested whether systemic colonisation of *A. thaliana* by the insect-pathogenic fungus *B. bassiana* induces resistance against insect and fungal plant antagonists. Furthermore, we investigated whether the resistance can be explained by correlational changes in gene expression, signalling molecules and secondary metabolites related to plant defences.

Our analysis of different plant parameters showed that growth promotion is strain-dependent as only BG11 but not FRh2 colonisation resulted in an increased number of leaves, shorter inflorescences and higher root biomass. Growth promotion by entomopathogens also varied in other studies and in some systems depended on the species or strain used as well as on soil nutrient availability (Jaber and Enkerli, 2017; Tall and Meyling, 2018). Gomez-Vidal et al. (2009) showed that *B. bassiana* can induce proteins related to photosynthesis and energy metabolism which could enhance plant growth. However, in the present study subsequent transcriptomic analyses did not indicate enhanced gene expression related to primary metabolism or photosynthesis and further research will be necessary to elucidate the underlying mechanisms of plant growth promotion by BG11.

Regarding the question whether the endophyte confers resistance against different plant antagonists, we observed that inoculation of *A. thaliana* roots with FRh2 and BG11, respectively, did not result in any negative effects on insect herbivores. Neither *B. bassiana* strain affected *P. xylostella* development or *M. persicae* population growth in this study and suggests that herbivore resistance is species-specific. This lack of effect could have been due to the low presence of *B. bassiana* after 4 weeks of endophytic growth, which was around 50% in the tested plant population. It should be noted that mortality of *P. xylostella* larvae in the controls was relatively high (29%) due to first instar losses. High baseline mortality could have been due to switching from *B. oleracea* to *A. thaliana* as a host plant at the start of the experiment. It is also conceivable that handling the small delicate insects with a soft paintbrush, despite taking great care, might have had some adverse effects. Baseline mortality of *P. xylostella* seems to vary considerably between 10 and 43% (Haas et al., 2018; Sun et al., 2018). Nevertheless, both endophyte strains caused a significant decrease in the area of necrotic leaf lesions by *S. sclerotiorum*. We suggest that, to some extent, this may be due to the induction of local and/or systemic plant resistance. The genome-wide expression analysis of *A. thaliana* provided evidence for the transcriptional reprogramming of plant defence pathways following *B. bassiana* colonisation, which may explain the observed effects against the pathogen. Interestingly, gene responses to both strains differed in several aspects, despite the fact that pathogen resistance was conferred by both *B. bassiana* fungi. We also found that, overall, the transcriptional responses to *B. bassiana* colonisation seemed to resemble those of other endophytic, non-entomopathogenic fungi, such as *Trichoderma* spp. and *Piriformospora indica* which are known inducers of systemic resistance against plant pathogens (Molitor et al., 2011; Mathys et al., 2012; Nongbri et al., 2012; Brotman et al., 2013; Gill et al., 2016).

Gene ontology and enrichment analyses suggested that both *B. bassiana* strains initiate microbial-associated molecular pattern triggered immunity (MTI) in *A. thaliana* as indicated by differential gene expression in the GO categories “response to innate immune response”, and “defence response” (Table 1 and Supplementary Tables S1–S4), suggesting that *B. bassiana* was recognised as an invading fungus. Both fungal strains induced a number of genes for receptor-like kinases, including a LysM domain-containing protein, which is necessary for chitin signalling and fungal resistance in *A. thaliana* (Wan et al., 2008). The induction of these genes may indicate that *Beauveria*-derived chitin is recognised by the plant as microbial-associated molecular pattern (MAMP) and could have contributed to enhanced resistance against *S. sclerotiorum*. Mathys et al. (2012) and Brotman et al. (2013) reported responses to chitin as one of the significantly upregulated biological processes in endophytic *Trichoderma* spp.–*A. thaliana* interactions. In addition, the upregulation of different pattern recognition receptors (PRRs) indicative for MTI was observed (Yang et al., 2012; Tang et al., 2017), such as the receptor-like kinase *FRK1* (in FRh2-inoculated plants) that is involved in the early defence response against the 22-amino-acid peptide Flg22 (Asai et al., 2002), and *AtRLP23* (in BG11-inoculated plants), which confers Nep1-like proteins (NLPs) recognition. These proteins are widely distributed among bacteria, fungi and oomycetes (Albert et al., 2015). WRKY transcription factors act as important regulators of complex defence networks and are central players of many aspects of MTI (Rushton et al., 2010; Pieterse et al., 2012). *WRKY29*, which was induced in FRh2-inoculated plants, is linked to enhanced resistance to bacterial and fungal pathogens (Asai et al., 2002) while *WRKY75*, induced in FRh2- and BG11-inoculated plants, is associated with defence responses against necrotrophic pathogens (Gechev et al., 2005; Encinas-Villarejo et al., 2009). In particular the induction of *WRKY75* could have played a role in the observed resistance against *S. sclerotiorum*, since Chen et al. (2013) showed that *WRKY75* overexpression lines conferred enhanced resistance to the same fungal species. In addition, FRh2- and BG11-inoculated plants were characterised by altered expression patterns of nucleotide binding site-leucine-rich repeat (NBS-LRR) genes, which act as plant immune receptors and are responsible for the initiation of a secondary line of plant defence called effector-triggered immunity (ETI). ETI is mediated by a complex recognition process of pathogen effectors by corresponding resistance proteins in the host plant (Jones and Dangl, 2006).

Downstream of MTI activation, different plant hormones such SA, JA, and ET are key players in the regulation of plant defence responses (Bari and Jones, 2009; Pieterse et al., 2012). GO analysis of the transcriptomic responses to BG11 suggested activation of the SA signalling pathway (“response to SA”), which is involved in systemic acquired resistance against pathogens and sucking herbivores such as aphids. Specifically, we observed the induction of SA marker genes such as *PR1*, *PR2*, *GRX480*, and *WRKY6* (Maleck et al., 2000; Robatzek and Somssich, 2002; Ndamukong et al., 2007). In addition, BG11 induced genes associated with the GO categories “response to JA”, “response to ET”, “response to wounding” and “JA-mediated signalling pathway” (Supplementary Tables S1–S4), namely *PDF1.2*, *ORA 59*, *ERF1*, *ERF2* and *WRKY 40* (Pre et al., 2008). This aligns with many studies that have shown similar effects of *Trichoderma* spp. colonisation on SA and JA–ET pathway activation (Salas-Marina et al., 2011; Mathys et al., 2012; Yoshioka et al., 2012; Brotman et al., 2013). A different picture emerged in the transcriptome of FRh2-inoculated plants: despite FRh2 inducing downregulation of JA–ET-regulated genes, this did not affect the expression of marker genes such as *PDF 1.2*. Similarly, FRh2 induced no effect on SA-pathway marker genes. The unaltered expression of SA and JA–ET related genes does not necessarily exclude the involvement of both pathways in the FRh2-triggered resistance against *S. sclerotiorum*. Using mutant analysis, Mathys et al. (2012) demonstrated the involvement of the JA-pathway in the *T. hamatum* T382–*A. thaliana* interaction, despite the fact that none of the JA-pathway marker genes were induced.

Following pathogen recognition and hormone signalling, plant immunity relies on the production of antimicrobial compounds, known as phytoalexins, that act against intrusive pathogens (Dodds and Rathjen, 2010). Both *B. bassiana* strains FRh2 and BG11 induced biosynthesis genes of the phytoalexin camalexin, such as *CYP71A12/13* and *CYP71B15* (*PAD3*), respectively. Similarly, Salas-Marina et al. (2011) reported an upregulation in *PAD3* expression in both roots and leaves after treatment of *A. thaliana* with *T. atroviride*, whereas Contreras-Cornejo et al. (2011) found that *A. thaliana* seedlings colonised by *T. virens* and *T. atroviride* accumulate camalexin in their leaves. It is possible that camalexin-induction or priming by *B. bassiana* could have resulted in enhanced resistance against *S. sclerotiorum*. Stotz et al. (2011) showed that *A. thaliana* genes associated with the formation of camalexin were induced in leaves challenged with *S. sclerotiorum*, whereas in mutant lines deficient in camalexin biosynthesis was hyper-susceptible to this phytopathogen.

Furthermore, *Beauveria*-colonised plants showed responses to oxidative stress that are likewise characteristic for *Trichoderma*–*A. thaliana* interactions (Salas-Marina et al., 2011; Mathys et al., 2012; Brotman et al., 2013). Multiple scavenger genes such as peroxidase, GST and other enzymes that protect against reactive oxygen species (ROS) were induced. A key regulator of the ascorbate-glutathione pathway for ROS detoxification (Yoon et al., 2004), the mono-dehydro-ascorbate reductase (MHDAR), was also upregulated in BG11-inoculated plants. This reductase is involved in the interaction between *A. thaliana* roots and the endophyte *P. indica* (Vadassery et al., 2009). ROS scavengers help plants not only to cope with biotic but also with abiotic stresses (Apel and Hirt, 2004; Daudi et al., 2012; Das and Roychoudhury, 2014). Interestingly, FRh2 colonisation differed from BG11 by triggering a stronger expression of various genes associated with abiotic stress. Changes in the expression of *CYP707A3, WRKY63, RAP2.6L, RAV2, HSP101* and *MBF1c* suggest that FRh2 may protect its host against drought or salinity (Umezawa et al., 2006; Krishnaswamy et al., 2011) and heat (Queitsch et al., 2000; Suzuki et al., 2008; Fu et al., 2014; Park and Seo, 2015). However, this hypothesis awaits further testing. Endophytes such as *Trichoderma* spp. and *P. indica* are well-known for their abilities to alleviate osmotic, salt, temperature or other abiotic stresses (Waller et al., 2005; Baltruschat et al., 2008; Brotman et al., 2013; Contreras-Cornejo et al., 2014).

The induction of SA and JA–ET dependent transcriptional responses in *A. thaliana* following BG11 colonisation did not result in increased levels of SA and JA metabolites in *A. thaliana* leaves. It confirms that gene expression does not necessarily reflect metabolite levels, which can be regulated at various post-expression stages (Pieterse et al., 2000; Van der Ent et al., 2009; Withers and Dong, 2017). However, this finding is in line with FRh2-inoculated plants, where genes of the defence pathways remained unaffected. Since both strains were able to enhance resistance against *S. sclerotiorum*, maybe signalling pathways other than those requiring SA and JA were involved. Future work, using SA and JA mutants of *A. thaliana*, will be needed to elucidate this question. At this point it is unclear whether two different resistance mechanisms have resulted in the same outcome or whether the plant responded similarly to both fungal strains with regard to pathogen defence. In any case, there is a certain overlap in the induction of SAR related genes, including, for instance ALD1, which plays a key role in local and systemic defence in *A. thaliana* and is involved in SA-independent signalling upstream of SA biosynthesis (Song et al., 2004; Návarová et al., 2012). This could point to a similar resistance mechanism in both cases.

As expected from the observed transcriptomic responses, *B. bassiana* colonisation did not result in major changes in the plant’s GLS profiles. Nevertheless, three AGS were attenuated in plants colonised by FRh2. Glucosinolates play an important role in the resistance of Brassicales against pathogens and insects (Hopkins et al., 2009; Wittstock and Burow, 2010; Buxdorf et al., 2013) and the fact that glucosinolates were not enhanced could explain to some extent why *B. bassiana* did not affect the performance of the generalist *M. persicae*, which has been shown to be susceptible to these compounds (Pfalz et al., 2009). Less likely GLS effects, on the other hand, would be expected in the case of *P. xylostella*, as this herbivore produces a sulfatase which prevents the formation of toxic GLS hydrolysis products (Ratzka et al., 2002). Regarding *S. sclerotiorum*, glucosinolates have been found to be induced by this fungus and also restricted pathogenesis (Stotz et al., 2011). Inoculation with *B. bassiana* may have either primed the plants to respond more strongly after *S. sclerotiorum* infection or other factors were important in this interaction. In a different endophyte system, Maag et al. (2014) likewise found no changes in GLS levels in *Brassica napus* after *T. atroviride* inoculation, and subsequent insect feeding did not lead to priming.

While this study provides correlational evidence for induced resistance against *S. sclerotiorum* based on the upregulation of pathogenesis-related (PR) proteins, ROS scavengers, camalexin, phytohormones and other defence-related genes, direct mechanisms that were not explored here cannot be ruled out entirely. Mycoparasitism within the plant tissue, competition for nutrients and toxic effects caused by *B. bassiana*-produced secondary metabolites could have exerted an inhibitory effect on the pathogen (Jaber and Ownley, 2018). However, evidence that such effects occur *in planta* is still missing.

In summary, our results demonstrate that *B. bassiana* protects *A. thaliana* against fungal disease but not against two herbivorous insect species. Endophytic colonisation resulted in profound changes in plant defence gene expression but in only few modifications in defence hormone and GLS levels. The presented study is one of the first to report plant responses to endophytic entomopathogens and contributes to a better understanding of *B. bassiana* ecology. The findings are also important for assessing the potential of endophytic entomopathogens as biological control agents.

## Data Availability

The datasets generated for this study can be found in Lincoln University Research Archive, https://researcharchive.lincoln.ac.nz/handle/10182/8097.

## Author Contributions

MRa, MRo, and TG conceived the study. MRa, HB, and CM carried out the experimental work. MRa, MRo, and HB analysed the data. MRa and MRo wrote the manuscript with contributions from all authors.

## Conflict of Interest Statement

The authors declare that the research was conducted in the absence of any commercial or financial relationships that could be construed as a potential conflict of interest.

## Footnotes

1 https://www.ncbi.nlm.nih.gov/tools/primer-blast/

2 https://CRAN.R-project.org

## References

[B1] Abdalsamee M. K. Müller C. (2012). Effects of indole glucosinolates on performance and sequestration by the sawfly *Athalia rosae* and consequences of feeding on the plant defense system. *J. Chem. Ecol.* 38 1366–1375. 10.1007/s10886-012-0197-4 23053922

[B2] Abel S. Nguyen M. D. Chow W. Theologis A. (1995). ASC4, a primary indoleacetic acid-responsive gene encoding 1-aminocyclopropane-1-carboxylate synthase in *Arabidopsis thaliana*. *J. Biol. Chem.* 270 19093–19099. 10.1074/jbc.270.32.19093 7642574

[B3] Albert I. Böhm H. Albert M. Feiler C. E. Imkampe J. Wallmeroth N. (2015). An RLP23–SOBIR1–BAK1 complex mediates NLP-triggered immunity. *Nat. Plants* 1:15140. 10.1038/nplants.2015.140 27251392

[B4] Apel K. Hirt H. (2004). Reactive oxygen species: metabolism, oxidative stress, and signal transduction. *Annu. Rev. Plant Biol.* 55 373–399. 10.1146/annurev.arplant.55.031903.141701 15377225

[B5] Asai T. Tena G. Plotnikova J. Willmann M. R. Chiu W. L. Gomez-Gomez L. (2002). MAP kinase signalling cascade in *Arabidopsis* innate immunity. *Nature* 415 977–983. 10.1038/415977a 11875555

[B6] Ashburner M. Ball C. A. Blake J. A. Botstein D. Butler H. Cherry J. M. (2000). Gene ontology: tool for the unification of biology. *Nat. Genet.* 25 25–29. 10.1038/75556 10802651 PMC3037419

[B7] Bakshi M. Oelmuller R. (2014). WRKY transcription factors: jack of many trades in plants. *Plant Signal. Behav.* 9:e27700. 10.4161/psb.27700 24492469 PMC4091213

[B8] Baltruschat H. Fodor J. Harrach B. D. Niemczyk E. Barna B. Gullner G. (2008). Salt tolerance of barley induced by the root endophyte *Piriformospora indica* is associated with a strong increase in antioxidants. *New Phytol.* 180 501–510. 10.1111/j.1469-8137.2008.02583.x 18681935

[B9] Bari R. Jones J. D. (2009). Role of plant hormones in plant defence responses. *Plant Mol. Biol.* 69 473–488. 10.1007/s11103-008-9435-0 19083153

[B10] Benjamini Y. Hochberg Y. (1995). Controlling the false discovery rate: a practical and powerful approach to multiple testing. *J. R. Stat. Soc. Ser. B* 57 289–300. 10.1111/j.2517-6161.1995.tb02031.x

[B11] Bing L. A. Lewis L. C. (1991). Suppression of *Ostrinia nubilalis* (Hubner) (Lepidoptera, Pyralidae) by endophytic *Beauveria bassiana* (Balsamo) Vuillemin. *Environ. Entomol.* 20 1207–1211. 10.1093/ee/20.4.1207

[B12] Biswas C. Dey P. Satpathy S. Satya P. (2012). Establishment of the fungal entomopathogen *Beauveria bassiana* as a season long endophyte in jute (*Corchorus olitorius*) and its rapid detection using SCAR marker. *Biocontrol* 57 565–571. 10.1007/s10526-011-9424-0

[B13] Bolstad B. M. Irizarry R. A. Astrand M. Speed T. P. (2003). A comparison of normalization methods for high density oligonucleotide array data based on variance and bias. *Bioinformatics* 19 185–193. 10.1093/bioinformatics/19.2.185 12538238

[B14] Brotman Y. Landau U. Cuadros-Inostroza A. Takayuki T. Fernie A. R. Chet I. (2013). *Trichoderma*-plant root colonization: escaping early plant defense responses and activation of the antioxidant machinery for saline stress tolerance. *PLoS Pathog.* 9:15. 10.1371/journal.ppat.1003221 23516362 PMC3597500

[B15] Brown P. D. Tokuhisa J. G. Reichelt M. Gershenzon J. (2003). Variation of glucosinolate accumulation among different organs and developmental stages of *Arabidopsis thaliana*. *Phytochemistry* 62 471–481. 10.1016/S0031-9422(02)00549-6 12620360

[B16] Brownbridge M. Reay S. D. Nelson T. L. Glare T. R. (2012). Persistence of *Beauveria bassiana* (Ascomycota: Hypocreales) as an endophyte following inoculation of radiata pine seed and seedlings. *Biol. Control* 61 194–200. 10.1016/j.biocontrol.2012.01.002

[B17] Buxdorf K. Yaffe H. Barda O. Levy M. (2013). The effects of glucosinolates and their breakdown products on necrotrophic fungi. *PLoS One* 8:e70771. 10.1371/journal.pone.0070771 23940639 PMC3733641

[B18] Card S. Johnson L. Teasdale S. Caradus J. (2016). Deciphering endophyte behaviour: the link between endophyte biology and efficacious biological control agents. *FEMS Microbiol. Ecol.* 92:fiw114. 10.1093/femsec/fiw114 27222223

[B19] Castrillo L. A. Vandenberg J. D. Wraight S. P. (2003). Strain-specific detection of introduced *Beauveria bassiana* in agricultural fields by use of sequence-characterized amplified region markers. *J. Invertebr. Pathol.* 82 75–83. 10.1016/S0022-2011(02)00190-8 12623307

[B20] Chen X. Liu J. Lin G. Wang A. Wang Z. Lu G. (2013). Overexpression of *AtWRKY28* and *AtWRKY75* in *Arabidopsis* enhances resistance to oxalic acid and *Sclerotinia sclerotiorum*. *Plant Cell Rep.* 32 1589–1599. 10.1007/s00299-013-1469-3 23749099

[B21] Contreras-Cornejo H. A. Macias-Rodriguez L. Alfaro-Cuevas R. Lopez-Bucio J. (2014). Trichoderma spp. improve growth of Arabidopsis seedlings under salt stress through enhanced root development, osmolite production, and Na+ elimination through root exudates. *Mol. Plant Microbe. Interact.* 27 503–514. 10.1094/MPMI-09-13-0265-R 24502519

[B22] Contreras-Cornejo H. A. Macias-Rodriguez L. Beltran-Pena E. Herrera-Estrella A. Lopez-Bucio J. (2011). Trichoderma-induced plant immunity likely involves both hormonal- and camalexin-dependent mechanisms in *Arabidopsis thaliana a*nd confers resistance against necrotrophic fungi *Botrytis cinerea*. *Plant Signal. Behav.* 6 1554–1563. 10.4161/psb.6.10.17443 21931272 PMC3256384

[B23] Das K. Roychoudhury A. (2014). Reactive oxygen species (ROS) and response of antioxidants as ROS-scavengers during environmental stress in plants. *Front. Environ. Sci.* 2:53 10.3389/fenvs.2014.00053

[B24] Daudi A. Cheng Z. O’Brien J. A. Mammarella N. Khan S. Ausubel F. M. (2012). The apoplastic oxidative burst peroxidase in *Arabidopsis* is a major component of pattern-triggered immunity. *Plant Cell* 24 275–287. 10.1105/tpc.111.093039 22247251 PMC3289579

[B25] Dodds P. N. Rathjen J. P. (2010). Plant immunity: towards an integrated view of plant–pathogen interactions. *Nat. Rev. Genet.* 11:539. 10.1038/nrg2812 20585331

[B26] Encinas-Villarejo S. Maldonado A. M. Amil-Ruiz F. de los Santos B. Romero F. Pliego-Alfaro F. (2009). Evidence for a positive regulatory role of strawberry (*Fragaria* x *ananassa*) Fa WRKY1 and *Arabidopsis* At WRKY75 proteins in resistance. *J. Exp. Bot.* 60 3043–3065. 10.1093/jxb/erp152 19470657

[B27] Frerigmann H. Gigolashvili T. (2014). MYB34, MYB51, and MYB122 distinctly regulate indolic glucosinolate biosynthesis in *Arabidopsis thaliana*. *Mol. Plant* 7 814–828. 10.1093/mp/ssu004 24431192

[B28] Fu M. Kang H. K. Son S. H. Kim S. K. Nam K. H. (2014). A subset of *Arabidopsis* RAV transcription factors modulates drought and salt stress responses independent of ABA. *Plant Cell Physiol.* 55 1892–1904. 10.1093/pcp/pcu118 25189341

[B29] Ganley R. J. Newcombe G. (2006). Fungal endophytes in seeds needles of *Pinus monticola*. *Mycol. Res.* 110 318–327. 10.1016/j.mycres.2005.10.005 16492396

[B30] Gechev T. S. Minkov I. N. Hille J. (2005). Hydrogen peroxide-induced cell death in *Arabidopsis*: transcriptional and mutant analysis reveals a role of an oxoglutarate-dependent dioxygenase gene in the cell death process. *IUBMB Life* 57 181–188. 10.1080/15216540500090793 16036580

[B31] Gill S. S. Gill R. Trivedi D. K. Anjum N. A. Sharma K. K. Ansari M. W. (2016). *Piriformospora indica*: potential and significance in plant stress tolerance. *Front. Microbiol.* 7:332 10.3389/fmicb.2016.00332 PMC4801890 27047458

[B32] Glare T. Caradus J. Gelernter W. Jackson T. Keyhani N. Kohl J. (2012). Have biopesticides come of age? *Trends Biotechnol.* 30 250–258. 10.1016/j.tibtech.2012.01.003 22336383

[B33] Golo P. S. Gardner D. R. Grilley M. M. Takemoto J. Y. Krasnoff S. B. Pires M. S. (2014). Production of destruxins from *Metarhizium* spp. fungi in artificial medium and in endophytically colonized cowpea plants. *PLoS One* 9:e104946. 10.1371/journal.pone.0104946 25127450 PMC4134251

[B34] Gomez-Vidal S. Salinas J. Tena M. Lopez-Llorca L. V. (2009). Proteomic analysis of date palm (*Phoenix dactylifera* L.) responses to endophytic colonization by entomopathogenic fungi. *Electrophoresis* 30 2996–3005. 10.1002/elps.200900192 19676091

[B35] Graser G. Oldham N. J. Brown P. D. Temp U. Gershenzon J. (2001). The biosynthesis of benzoic acid glucosinolate esters in *Arabidopsis thaliana*. *Phytochemistry* 57 23–32. 10.1016/S0031-9422(00)00501-X 11336257

[B36] Haas J. Lozana E. R. Haida K. S. Mazara S. M. Souza Vismara E. Poppy G. M. (2018). Getting ready for battle: do cabbage seeds acid treated with jasmonic sap chitosan affect chewing and-feeding insects? *Entomol. Exp. Appl*. 166 412–419. 10.1111/eea.12678

[B37] Hajek A. (2004). *Natural Enemies An Introduction to Biological Control.* Cambridge: Cambridge University Press 10.1017/CBO9780511811838

[B38] Hardtke C. S. Dorcey E. Osmont K. S. Sibout R. (2007). Phytohormone collaboration: zooming in on auxin–brassinosteroid interactions. *Trends Cell Biol.* 17 485–492. 10.1016/j.tcb.2007.08.003 17904848

[B39] Hermosa R. Viterbo A. Chet I. Monte E. (2012). Plant-beneficial effects of *Trichoderma* and of its genes. *Microbiology* 158 17–25. 10.1099/mic.0.052274-0 21998166

[B40] Hopkins R. J. van Dam N. M. van Loon J. J. A. (2009). Role of glucosinolates in insect-plant relationships and multitrophic interactions. *Annu. Rev. Entomol.* 54 57–83. 10.1146/annurev.ento.54.110807.090623 18811249

[B41] Jaber L. R. Enkerli J. (2017). Fungal entomopathogens as endophytes: can they promote plant growth? *Biocontrol Sci. Technol.* 27 28–41. 10.1080/09583157.2016.1243227

[B42] Jaber L. R. Ownley B. H. (2018). Can we use entomopathogenic fungi as endophytes for dual biological control of insect pests and plant pathogens? *Biol. Control* 116 36–45. 10.1016/j.jip.2015.07.009 26225455

[B43] Jones J. D. G. Dangl J. L. (2006). The plant immune system. *Nature* 444 323–329. 10.1038/nature05286 17108957

[B44] Kieber J. J. Schaller G. E. (2014). Cytokinins. *Arabidopsis Book* 12:e0168. 10.1199/tab.0168 24465173 PMC3894907

[B45] Krishnaswamy S. Verma S. Rahman M. H. Kav N. N. (2011). Functional characterization of four APETALA2-family genes (*RAP2.6, RAP2.6L, DREB19* and *DREB26*) in *Arabidopsis*. *Plant Mol. Biol.* 75 107–127. 10.1007/s11103-010-9711-7 21069430

[B46] Lacey L. A. Grzywacz D. Shapiro-Ilan D. I. Frutos R. Brownbridge M. Goettel M. S. (2015). Insect pathogens as biological control agents: back to the future. *J. Invertebr. Pathol.* 132 1–41. 10.1016/j.jip.2015.07.009 26225455

[B47] Lee D. S. Kim B. K. Kwon S. J. Jin H. C. Park O. K. (2009). *Arabidopsis* GDSL lipase 2 plays a role in pathogen defense via negative regulation of auxin signaling. *Biochem. Biophys. Res. Commun.* 379 1038–1042. 10.1016/j.bbrc.2009.01.006 19146828

[B48] Livak K. J. Schmittgen T. D. (2001). Analysis of relative gene expression data using real-time quantitative PCR and the 2(-Delta Delta C(T)) Method. *Methods* 25 402–408. 10.1006/meth.2001.1262 11846609

[B49] Lugtenberg B. J. J. Caradus J. R. Johnson L. J. (2016). Fungal endophytes for sustainable crop production. *FEMS Microbiol. Ecol.* 92:fiw194. 10.1093/femsec/fiw194 27624083

[B50] Maag D. Kandula D. R. W. Müller C. Mendoza-Mendoza A. Wratten S. D. Stewart A. (2014). *Trichoderma atroviride* LU132 promotes plant growth but not induced systemic resistance to *Plutella xylostella* in oilseed rape. *BioControl* 59 241–252. 10.1007/s10526-013-9554-7

[B51] Maleck K. Levine A. Eulgem T. Morgan A. Schmid J. Lawton K. A. (2000). The transcriptome of *Arabidopsis thaliana* during systemic acquired resistance. *Nat. Genet.* 26 403–410. 10.1038/82521 11101835

[B52] Mathys J. De Cremer K. Timmermans P. Van Kerckhove S. Lievens B. Vanhaecke M. (2012). Genome-wide characterization of ISR induced in *Arabidopsis thaliana* by *Trichoderma hamatum* T382 against *Botrytis cinerea* infection. *Front. Plant Sci.* 3:108. 10.3389/fpls.2012.00108 22661981 PMC3362084

[B53] McKinnon A. C. Saari S. Moran-Diez M. E. Meyling N. V. Raad M. Glare T. R. (2017). *Beauveria bassiana* as an endophyte: a critical review on associated methodology and biocontrol potential. *BioControl* 62 1–17. 10.1007/s10526-016-9769-5

[B54] Mi H. Huang X. Muruganujan A. Tang H. Mills C. Kang D. (2017). PANTHER version 11: expanded annotation data from Gene Ontology and Reactome pathways, and data analysis tool enhancements. *Nucleic Acids Res.* 45 D183–D189. 10.1093/nar/gkw1138 27899595 PMC5210595

[B55] Molitor A. Zajic D. Voll L. M. Pons K. H. J. Samans B. Kogel K. H. (2011). Barley leaf transcriptome and metabolite analysis reveals new aspects of compatibility and *Piriformospora indica*-mediated systemic induced resistance to powdery mildew. *Mol. Plant Microbe Interact.* 24 1427–1439. 10.1094/MPMI-06-11-0177 21830949

[B56] Mukherjee P. K. Horwitz B. A. Herrera-Estrella A. Schmoll M. Kenerley C. M. (2013). *Trichoderma* research in the genome era. *Annu. Rev. Phytopathol.* 51 105–129. 10.1146/annurev-phyto-082712-102353 23915132

[B57] Muvea A. M. Meyhöfer R. Subramanian S. Poehling H.-M. Ekesi S. Maniana N. K. (2014). Colonisation of onions by endophytic fungi and their impacts on the biology of *Thrips tabaci*. *PLoS One* 9:e108242. 10.1371/journal.pone.0108242 25254657 PMC4177896

[B58] Návarová H. Bernsdorff F. Döring A.-C. Zeier J. (2012). Pipecolic acid, an endogenous mediator of defense amplification and priming, is a critical regulator of inducible plant immunity. *Plant Cell* 24 5123–5141. 10.1105/tpc.112.103564 23221596 PMC3556979

[B59] Ndamukong I. Abdallat A. A. Thurow C. Fode B. Zander M. Weigel R. (2007). SA-inducible *Arabidopsis* glutaredoxin interacts with TGA factors and suppresses JA-responsive *PDF1.2 transcription*. *Plant J.* 50 128–139. 10.1111/j.1365-313X.2007.03039.x 17397508

[B60] Nongbri P. Vahabi K. Mrozinska A. Seebald E. Sun C. Sherameti I. (2012). Balancing defense and growth—Analyses of the beneficial symbiosis between *Piriformospora indica* and *Arabidopsis thaliana*. *Symbiosis* 58 17–28. 10.1007/s13199-012-0209-8

[B61] Oh I. S. Park A. R. Bae M. S. Kwon S. J. Kim Y. S. Lee J. E. (2005). Secretome analysis reveals an *Arabidopsis* lipase involved in defense against *Alternaria brassicicola*. *Plant Cell* 17 2832–2847. 10.1105/tpc.105.034819 16126835 PMC1242276

[B62] Ownley B. H. Griffin M. R. Klingeman W. E. Gwinn K. D. Moulton J. K. Pereira R. M. (2008). *Beauveria bassiana*: endophytic colonization and plant disease control. *J. Invertebr. Pathol.* 98 267–270. 10.1016/j.jip.2008.01.010 18442830

[B63] Park C. J. Seo Y. S. (2015). Heat shock proteins: a review of the molecular chaperones for plant immunity. *Plant Pathol. J.* 31 323–333. 10.5423/PPJ.RW.08.2015.0150 26676169 PMC4677741

[B64] Pfalz M. Vogel H. Kroymann J. (2009). The gene controlling the *Indole Glucosinolate Modifier1* quantitative trait locus alters indole glucosinolate structures and aphid resistance in *Arabidopsis*. *Plant Cell* 21 985–999. 10.1105/tpc.108.063115 19293369 PMC2671713

[B65] Pieterse C. M. Zamioudis C. Berendsen R. L. Weller D. M. Van Wees S. C. Bakker P. A. (2014). Induced systemic resistance by beneficial microbes. *Annu. Rev. Phytopathol.* 52 347–375. 10.1146/annurev-phyto-082712-102340 24906124

[B66] Pieterse C. M. J. Van der Does D. Zamioudis C. Leon-Reyes A. Van Wees S. C. (2012). Hormonal modulation of plant immunity. *Annu. Rev. Cell. Dev. Biol.* 28 489–521. 10.1146/annurev-cellbio-092910-154055 22559264

[B67] Pieterse C. M. J. Van Pelt J. A. Ton J. Parchmann S. Mueller M. J. Buchala A. J. (2000). *Rhizobacteria*-mediated induced systemic resistance (ISR) in *Arabidopsis* requires sensitivity to jasmonate and ethylene but is not accompanied by an increase in their production. *Physiol. Mol. Plant Pathol.* 57 123–134. 10.1006/pmpp.2000.0291

[B68] Pre M. Atallah M. Champion A. De Vos M. Pieterse C. M. J. Memelink J. (2008). The AP2/ERF domain transcription factor ORA59 integrates jasmonic acid and ethylene signals in plant defense. *Plant Physiol.* 147 1347–1357. 10.1104/pp.108.117523 18467450 PMC2442530

[B69] Qayyum M. A. Wakil W. Arif M. J. Sahi S. T. Dunlap C. A. (2015). Infection of *Helicoverpa armigera* by endophytic *Beauveria bassiana* colonizing tomato plants. *Biol. Control* 90 200–207. 10.1016/j.biocontrol.2015.04.005

[B70] Queitsch C. Hong S. W. Vierling E. Lindquist S. (2000). Heat shock protein 101 plays a crucial role in thermotolerance in *Arabidopsis*. *Plant Cell* 12 479–492. 10.1105/tpc.12.4.479 10760238 PMC139847

[B71] Ramos Y. Portal O. Lysoe E. Meyling N. V. Klingen I. (2017). Diversity and abundance of *Beauveria bassiana* in soils, stink bugs and plant tissues of common bean from organic and conventional fields. *J. Invertebr. Pathol.* 150 114–120. 10.1016/j.jip.2017.10.003 29042323

[B72] Ratzka A. Vogel H. Kliebenstein D. J. Mitchell-Olds T. Kroymann J. (2002). Disarming the mustard oil bomb. *Proc. Natl. Acad. Sci. U.S.A.* 99 11223–11228. 10.1073/pnas.172112899 12161563 PMC123237

[B73] Reay S. D. Brownbridge M. Gicquel B. Cummings N. J. Nelson T. L. (2010). Isolation and characterization of endophytic *Beauveria* spp. (*Ascomycota: Hypocreales*) from Pinus radiata in New Zealand forests. *Biol. Control* 54 52–60. 10.1016/j.biocontrol.2010.03.002

[B74] Ren X. Chen Z. Liu Y. Zhang H. Zhang M. Liu Q. (2010). ABO3, a WRKY transcription factor, mediates plant responses to abscisic acid and drought tolerance in *Arabidopsis*. *Plant J.* 63 417–429. 10.1111/j.1365-313X.2010.04248.x 20487379 PMC3117930

[B75] Robatzek S. Somssich I. E. (2002). Targets of *AtWRKY6* regulation during plant senescence and pathogen defense. *Genes Dev.* 16 1139–1149. 10.1101/gad.222702 12000796 PMC186251

[B76] Rostás M. Ton J. Mauch-Mani B. Turlings T. C. (2006). Fungal infection reduces herbivore-induced plant volatiles of maize but does not affect naive parasitoids. *J. Chem. Ecol.* 32 1897–1909. 10.1007/s10886-006-9147-3 16902818

[B77] Roy H. E. Steinkraus D. C. Eilenberg J. Hajek A. E. Pell J. K. (2006). Bizarre interactions and endgames: entomopathogenic fungi and their arthropod hosts. *Annu. Rev. Entomol.* 51 331–357. 10.1146/annurev.ento.51.110104.150941 16332215

[B78] Rushton P. J. Somssich I. E. Ringler P. Shen Q. J. (2010). WRKY transcription factors. *Trends Plant Sci.* 15 247–258. 10.1016/j.tplants.2010.02.006 20304701

[B79] Salas-Marina M. A. Silva-Flores M. A. Uresti-Rivera E. E. Castro-Longoria E. Herrera-Estrella A. Casas-Flores S. (2011). Colonization of *Arabidopsis* roots by *Trichoderma atroviride* promotes growth and enhances systemic disease resistance through jasmonic acid/ethylene and salicylic acid pathways. *Eur. J. Plant Pathol.* 131 15–26. 10.1007/s10658-011-9782-6

[B80] Schmelz E. A. Engelberth J. Tumlinson J. H. Block A. Alborn H. T. (2004). The use of vapor phase extraction in metabolic profiling of phytohormones and other metabolites. *Plant J.* 39 790–808. 10.1111/j.1365-313X.2004.02168.x 15315639

[B81] Schulz B. Boyle C. (2005). The endophytic continuum. *Mycol. Res.* 109 661–686. 10.1017/S095375620500273X 16080390

[B82] Song J. T. Lu H. McDowell J. M. Greenberg J. T. (2004). A key role for ALD1 in activation of local and systemic defenses in Arabidopsis. *Plant J.* 40 200–212. 10.1111/j.1365-313X.2004.02200.x 15447647

[B83] Steyaert J. M. Ridgway H. J. Elad Y. Stewart A. (2003). Genetic basis of mycoparasitism: a mechanism of biological control by species of *Trichoderma*. *N. Z. J. Crop Hortic. Sci.* 31 281–291. 10.1080/01140671.2003.9514263

[B84] Stotz H. U. Sawada Y. Shimada Y. Hirai M. Y. Sasaki E. Krischke M. (2011). Role of camalexin, indole glucosinolates, and side chain modification of glucosinolate-derived isothiocyanates in defense of *Arabidopsis* against *Sclerotinia sclerotiorum*. *Plant J.* 67 81–93. 10.1111/j.1365-313X.2011.04578.x 21418358

[B85] Sun B. T. Akutse K. S. Xia X. F. Chen J. H. Ai X. Tang Y. (2018) Endophytic effects of *Aspergillus oryzae* on radish (*Raphanus sativus*) and its herbivore, *Plutella xylostella*. *Planta* 248 705–714. 10.1007/s00425-018-2928-4 29948125

[B86] Suzuki N. Bajad S. Shuman J. Shulaev V. Mittler R. (2008). The transcriptional co-activator MBF1c is a key regulator of thermotolerance in *Arabidopsis thaliana*. *J. Biol. Chem.* 283 9269–9275. 10.1074/jbc.M709187200 18201973

[B87] Sword G. A. Tessnow A. Ek-Ramos M. J. (2017). Endophytic fungi alter sucking bug responses to cotton reproductive structures. *Insect Sci.* 24 1003–1014. 10.1111/1744-7917.12461 28328087

[B88] Tall S. Meyling N. V. (2018). Probiotics for plants? growth promotion by the entomopathogenic fungus *beauveria bassiana* depends on nutrient availability. *Microbe. Ecol.* 76 1002–1008. 10.1007/s00248-018-1180-6 29594431

[B89] Tang D. Wang G. Zhou J.-M. (2017). Receptor kinases in plant-pathogen interactions: more than pattern recognition. *Plant Cell* 29 618–637. 10.1105/tpc.16.00891 28302675 PMC5435430

[B90] The Gene Ontology Consortium (2017). Expansion of the gene ontology knowledgebase and resources. *Nucleic Acids Res.* 45 D331–D338. 10.1093/nar/gkw1108 27899567 PMC5210579

[B91] Thimm O. Blasing O. Gibon Y. Nagel A. Meyer S. Kruger P. (2004). MAPMAN: a user-driven tool to display genomics data sets onto diagrams of metabolic pathways and other biological processes. *Plant J.* 37 914–939. 10.1111/j.1365-313X.2004.02016.x 14996223

[B92] Umezawa T. Okamoto M. Kushiro T. Nambara E. Oono Y. Seki M. (2006). CYP707A3, a major ABA 8’-hydroxylase involved in dehydration and rehydration response in *Arabidopsis thaliana*. *Plant J.* 46 171–182. 10.1111/j.1365-313X.2006.02683.x 16623881

[B93] Usadel B. Poree F. Nagel A. Lohse M. Czedik-Eysenberg A. Stitt M. (2009). A guide to using MapMan to visualize and compare Omics data in plants: a case study in the crop species, Maize. *Plant Cell Environ.* 32 1211–1229. 10.1111/j.1365-3040.2009.01978.x 19389052

[B94] Vadassery J. Tripathi S. Prasad R. Varma A. Oelmuller R. (2009). Monodehydroascorbate reductase 2 and dehydroascorbate reductase 5 are crucial for a mutualistic interaction between *Piriformospora indica* and *Arabidopsis*. *J. Plant Physiol.* 166 1263–1274. 10.1016/j.jplph.2008.12.016 19386380

[B95] Van der Ent S. Van Wees S. C. M. Pieterse C. M. J. (2009). Jasmonate signaling in plant interactions with resistance-inducing beneficial microbes. *Phytochemistry* 70 1581–1588. 10.1016/j.phytochem.2009.06.009 19712950

[B96] Vega F. E. (2018). The use of fungal entomopathogens as endophytes in biological control: a review. *Mycologia* 110 4–30. 10.1080/00275514.2017.1418578 29863999

[B97] Vega F. E. Goettel M. S. Blackwell M. Chandler D. Jackson M. A. Keller S. (2009). Fungal entomopathogens: new insights on their ecology. *Fungal Ecol.* 2 149–159. 10.1016/j.funeco.2009.05.001

[B98] Vega F. E. Simpkins A. Aime M. C. Posada F. Peterson S. W. Rehner S. A. (2010). Fungal endophyte diversity in coffee plants from Colombia, Hawai’i, Mexico and Puerto Rico. *Fungal Ecol.* 3 122–138. 10.1016/j.funeco.2009.07.002 16187260

[B99] Vidal S. Jaber L. R. (2015). Entomopathogenic fungi as endophytes: plant-endophyte-herbivore interactions and prospects for use in biological control. *Curr. Sci.* 109 46–54.

[B100] Vos C. M. F. De Cremer K. Cammue B. P. A. De Coninck B. (2015). The toolbox of *Trichoderma* spp. in the biocontrol of Botrytis cinerea disease. *Mol. Plant Pathol.* 16 400–412. 10.1111/mpp.12189 25171761 PMC6638538

[B101] Waller F. Achatz B. Baltruschat H. Fodor J. Becker K. Fischer M. (2005). The endophytic fungus *Piriformospora indica* reprograms barley to salt-stress tolerance, disease resistance, and higher yield. *Proc. Natl. Acad. Sci. U.S.A.* 102 13386–13391. 10.1073/pnas.0504423102 16174735 PMC1224632

[B102] Wan J. Zhang X. C. Neece D. Ramonell K. M. Clough S. Kim S. Y. (2008). A LysM receptor-like kinase plays a critical role in chitin signaling and fungal resistance in *Arabidopsis*. *Plant Cell* 20 471–481. 10.1105/tpc.107.056754 18263776 PMC2276435

[B103] Withers J. Dong X. (2017). Post-translational regulation of plant immunity. *Curr. Opin. Plant Biol.* 38 124–132. 10.1016/j.pbi.2017.05.004 28538164 PMC5644497

[B104] Wittstock U. Burow M. (2010). Glucosinolate breakdown in *Arabidopsis*: mechanism, regulation and biological significance. *Arabidopsis Book* 8:e0134. 10.1199/tab.0134 22303260 PMC3244901

[B105] Yang X. Deng F. Ramonell K. M. (2012). Receptor-like kinases and receptor-like proteins: keys to pathogen recognition and defense signaling in plant innate immunity. *Front. Biol.* 7:155–166. 10.1007/s11515-011-1185-8

[B106] Yoon H. S. Lee H. Lee I. A. Kim K. Y. Jo J. (2004). Molecular cloning of the monodehydroascorbate reductase gene from *Brassica campestris* and analysis of its mRNA level in response to oxidative stress. *Biochim. Biophys. Acta* 1658 181–186. 10.1016/j.bbabio.2004.05.013 15450955

[B107] Yoshioka Y. Ichikawa H. Naznin H. A. Kogure A. Hyakumachi M. (2012). Systemic resistance induced in *Arabidopsis thaliana* by *Trichoderma asperellum* SKT-1, a microbial pesticide of seedborne diseases of rice. *Pest Manag. Sci.* 68 60–66. 10.1002/ps.2220 21674754

