# Corrigendum: Transcriptional Reprogramming of *Arabidopsis thaliana* Defence Pathways by the Entomopathogen *Beauveria bassiana* Correlates With Resistance Against a Fungal Pathogen but Not Against Insects

**DOI:** 10.3389/fmicb.2019.01481

**Published:** 2019-06-27

**Authors:** Maya Raad, Travis R. Glare, Helena L. Brochero, Caroline Müller, Michael Rostás

**Affiliations:** ^1^Bio-Protection Research Centre, Lincoln University, Lincoln, New Zealand; ^2^Facultad de Ciencias Agrarias, Universidad Nacional de Colombia, Bogotá, Colombia; ^3^Department of Chemical Ecology, Bielefeld University, Bielefeld, Germany; ^4^Department of Crop Sciences, Agricultural Entomology, University of Göttingen, Göttingen, Germany

**Keywords:** endophytes, glucosinolates, induced resistance, phytohormones, plant–microbe interaction, *Plutella xylostella*, *Myzus persicae*, *Sclerotinia sclerotiorum*

In the published article, there was an error in affiliation “2.” Instead of “Faculty of Agricultural Sciences, National University of Colombia, Bogotá, Colombia,” it should be “Facultad de Ciencias Agrarias, Universidad Nacional de Colombia, Bogotá, Colombia.”

The authors apologize for this error and state that this does not change the scientific conclusions of the article in any way. The original article has been updated.

