# Supplementary material for: Transcriptional Reprogramming of Arabidopsis thaliana Defence Pathways by the Entomopathogen Beauveria bassiana Correlates With Resistance Against a Fungal Pathogen but Not Against Insects
Source: Front Microbiol. 2019 Mar 29;10:615. doi: 10.3389/fmicb.2019.00615 (PMC6449843; doi:10.3389/fmicb.2019.00615)
Supplement: Supplementary file 1 [file Table_1.docx]

Table S1 Upregulated genes in FRh2-inoculated plants; differentially expressed biological processes enriched. (*p*-value < 0.05). Categories in bold and italic represent parents of GO terms.

| **GO biological process complete** | ***p*-value** |
| --- | --- |
|  |  |
| ***Response to hydrogen peroxide*** | ***1.15E-06*** |
| Response to inorganic substance | 3.26E-02 |
| Response to chemical | 1.05E-03 |
| Response to stimulus | 1.24E-05 |
| Response to reactive oxygen species | 5.18E-06 |
| Response to oxidative stress | 1.33E-04 |
| Response to stress | 9.56E-07 |
|  |  |
| ***Systemic acquired resistance*** | ***1.03E-02*** |
| Defence response, incompatible interaction | 4.06E-04 |
| Innate immune response | 1.41E-05 |
| Immune response | 2.04E-05 |
| Immune system process | 5.32E-06 |
| Defence response | 2.23E-03 |
| Defence response to other organism | 8.94E-05 |
| Response to other organism | 1.51E-05 |
| Response to external biotic stimulus | 1.60E-05 |
| Response to external stimulus | 8.32E-05 |
| Response to biotic stimulus | 5.12E-05 |
| Multi-organism process | 3.16E-02 |
|  |  |
| ***Response to heat*** | ***1.03E-06*** |
| Response to temperature stimulus | 6.47E-03 |
|  |  |
| ***Defence response to bacterium*** | ***2.48E-05*** |
| *Response to bacterium* | 1.80E-05 |
|  |  |
| ***Secondary metabolic process*** | ***2.61E-02*** |
|  |  |
| ***Unclassified*** | ***0.00E00*** |
|  |  |
| ***Cellular component organization*** | ***2.61E-02*** |
|  |  |
| Cellular component organization or biogenesis | 8.39E- |
