# Supplementary material for: Transcriptional Reprogramming of Arabidopsis thaliana Defence Pathways by the Entomopathogen Beauveria bassiana Correlates With Resistance Against a Fungal Pathogen but Not Against Insects
Source: Front Microbiol. 2019 Mar 29;10:615. doi: 10.3389/fmicb.2019.00615 (PMC6449843; doi:10.3389/fmicb.2019.00615)
Supplement: Supplementary file 2 [file Table_2.docx]

Table S2 Downregulated genes in FRh2-inoculated plants; differentially expressed biological processes enriched. (*p*-value < 0.05). (*p*-value < 0.05). Categories in bold and italic represent parents of GO terms.

| **GO biological process complete** | ***p*-value** |
| --- | --- |
|  |  |
| ***Xyloglucan metabolic process*** | ***9.45E-04*** |
| Hemicellulose metabolic process | 7.93E-03 |
| Cell wall polysaccharide metabolic process | 2.95E-02 |
|  |  |
| ***Gibberellic acid mediated signalling pathway*** | ***2.78E-02*** |
| Gibberellin mediated signalling pathway | 3.99E-02 |
| Hormone-mediated signalling pathway | 1.80E-06 |
| Cellular response to hormone stimulus | 7.79E-07 |
| Cellular response to endogenous stimulus | 1.08E-06 |
| Response to endogenous stimulus | 6.81E-03 |
| Cellular response to organic substance | 1.37E-05 |
| Cellular response to chemical stimulus | 8.90E-05 |
| Cellular response to stimulus | 2.52E-02 |
| Response to hormone | 5.76E-03 |
| Signal transduction | 9.22E-03 |
| Single organism signalling | 1.60E-02 |
| Signalling | 1.64E-02 |
| Cellular response to gibberellin stimulus | 4.48E-02 |
|  |  |
| ***Auxin-activated signalling pathway*** | ***2.39E-02*** |
| Cellular response to auxin stimulus | 2.36E-03 |
| Response to auxin | 7.62E-04 |
|  |  |
| ***External encapsulating structure organization*** | ***4.51E-02*** |
|  |  |
| ***Unclassified*** | ***0.00E00*** |
