# Supplementary material for: Transcriptional Reprogramming of Arabidopsis thaliana Defence Pathways by the Entomopathogen Beauveria bassiana Correlates With Resistance Against a Fungal Pathogen but Not Against Insects
Source: Front Microbiol. 2019 Mar 29;10:615. doi: 10.3389/fmicb.2019.00615 (PMC6449843; doi:10.3389/fmicb.2019.00615)
Supplement: Supplementary file 3 [file Table_3.docx]

Table S3 Upregulated genes in BG11-inoculated plants; differentially expressed biological processes enriched. (*p*-value < 0.05). Categories in bold and italic represent parents of GO terms.

| **GO biological process complete** | ***p*-value** |
| --- | --- |
|  |  |
| ***Regulation of systemic acquired resistance*** | ***4.17E-02*** |
| Regulation of response to external stimulus | 1.74E-02 |
| Regulation of innate immune response | 1.95E-03 |
| Regulation of immune response | 2.55E-03 |
| Regulation of immune system process | 4.36E-04 |
| Regulation of defence response | 1.01E-02 |
|  |  |
| ***Defence response, incompatible interaction*** | ***6.93E-06*** |
| Innate immune response | 8.70E-06 |
| Immune response | 1.15E-05 |
| Response to stimulus | 3.49E-07 |
| Immune system process | 5.26E-05 |
| Defence response | 3.77E-09 |
| Response to stress | 3.54E-12 |
| Defence response to other organism | 4.12E-04 |
| Response to other organism | 2.12E-08 |
| Response to external biotic stimulus | 2.25E-08 |
| Response to external stimulus | 9.12E-07 |
| Response to biotic stimulus | 6.48E-08 |
| Multi-organism process | 3.43E-06 |
|  |  |
| ***Response to jasmonic acid*** | ***1.76E-07*** |
| Response to acid chemical | 2.01E-05 |
| Response to chemical | 3.43E-07 |
| Response to oxygen-containing compound | 2.12E-06 |
| Response to hormone | 1.04E-03 |
| Response to organic substance | 7.98E-07 |
| Response to endogenous stimulus | 1.18E-03 |
|  |  |
| ***Response to wounding*** | ***2.39E-04*** |
|  |  |
| ***Response to salicylic acid*** | ***3.27E-03*** |
| Response to organic cyclic compound | 2.97E-03 |
|  |  |
| ***Response to ethylene*** | ***3.10E-03*** |
| *Table S3 continued* |  |
|  |  |
| ***Response to bacterium*** | ***7.23E-03*** |
|  |  |
| ***Response to oxidative stress*** | ***1.00E-02*** |
|  |  |
| ***Unclassified*** | ***0.00E00*** |
