# Supplementary material for: Transcriptional Reprogramming of Arabidopsis thaliana Defence Pathways by the Entomopathogen Beauveria bassiana Correlates With Resistance Against a Fungal Pathogen but Not Against Insects
Source: Front Microbiol. 2019 Mar 29;10:615. doi: 10.3389/fmicb.2019.00615 (PMC6449843; doi:10.3389/fmicb.2019.00615)
Supplement: Supplementary file 4 [file Table_4.docx]

Table S4 Downregulated genes in FRh2-inoculated plants; differentially expressed biological processes enriched. (*p*-value < 0.05). Categories in bold and italic represent parents of GO terms.

| **GO biological process complete** | | ***p*-value** |
| --- | --- | --- |
|  | |  |
| ***Cellular response to blue light*** | | ***2.24E-03*** |
| *Cellular response to abiotic stimulus* | | 9.00E-03 |
|  | |  |
| ***Unclassified*** |  | ***0.00E00*** |
