# Supplementary material for: Transcriptional Reprogramming of Arabidopsis thaliana Defence Pathways by the Entomopathogen Beauveria bassiana Correlates With Resistance Against a Fungal Pathogen but Not Against Insects
Source: Front Microbiol. 2019 Mar 29;10:615. doi: 10.3389/fmicb.2019.00615 (PMC6449843; doi:10.3389/fmicb.2019.00615)
Supplement: Supplementary file 5 [file Table_5.docx]

Table S5 Differentially expressed genes (DEGs) shared between AtFRh2 and AtBG11 (see also Fig. 3).

Red and blue boxes show up- and downregulated DEGs, respectively. Expression levels for each DEG shown as LogFC= log2 fold change >1 and <-1. AtFRh2 = DEGs resulting from the comparative transcriptome study for AtCO_vs_AtFRh2 and AtBG11 = DEGs resulting from the comparative transcriptome study for AtCO_vs_AtBG11.

|  |  | **LogFC** | |
| --- | --- | --- | --- |
| **ID** | **Symbols/Description** | **AtFRh2** | **AtBG11** |
| at1g61560 | MLO6 | 1.017 | 1.041 |
| at4g18430 | RABA1e | 1.101 | 1.059 |
| at1g33030 | O-methyltransferase family 2 protein | 1.121 | 1.001 |
| at1g61800 | GPT2 | 1.125 | 1.53 |
| at2g18660 | EXLB3 | 1.166 | 1.178 |
| at2g25440 | AtRLP20 | 1.206 | 1.178 |
| at1g69930 | GSTU11 | 1.232 | 1.226 |
| at1g04980 | PDIL2-2 | 1.253 | 1.007 |
| at4g25110 | AtMC2 | 1.297 | 1.008 |
| at3g61390 | U-box domain-containing protein | 1.314 | 1.351 |
| at5g62150 | LysM domain-containing protein | 1.331 | 1.141 |
| at4g09300 | unknown protein | 1.386 | 1.053 |
| at5g13080 | WRKY75 | 1.403 | 1.663 |
| at5g22530 | unknown protein | 1.475 | 1.004 |
| at4g25070 | unknown protein | 1.501 | 1.006 |
| at1g36640 | unknown protein | 1.633 | 1.335 |
| at5g67310 | CYP81G1 | 1.64 | 1.453 |
| at1g47890 | RLP7 | 1.678 | 1.331 |
| at1g21310 | EXT3, | 1.694 | 1.312 |
| at1g66960 | lupeol synthase, putative | 1.765 | 1.054 |
| at2g13810 | ALD1 | 1.801 | 1.785 |
| at1g21240 | WAK3 | 1.807 | 1.178 |
| at3g28580 | AAA-type ATPase family protein | 1.842 | 1.034 |
| at5g38900 | DSBA oxidoreductase family protein | 1.896 | 1.002 |
| at3g22600 | lipid transfer protein (LTP) family protein | 1.995 | 1.248 |
| at2g43570 | chitinase, putative | 1.998 | 1.374 |
| at3g63380 | calcium-transporting ATPase,putative | 2.004 | 1.661 |
| at4g11170 | disease resistance protein, putative | 2.2 | 1.124 |
| at5g11210 | GLR2.5 | 2.371 | 1.06 |
| at4g23700 | CHX17 | 2.409 | 1.002 |
| at1g44130 | nucellin protein, putative | 2.51 | 1.429 |
| at3g11340 | UDP-glucoronosyl family protein | 2.595 | 1.801 |
| at1g19250 | SFMO1 | 2.982 | 1.648 |
| at5g45920 | carboxylesterase/ hydrolase | -1.321 | -1.32 |
| at5g63160 | BT1 | -1.141 | -1.711 |
| at3g48115 | other RNA | -1.201 | -1.073 |
| at4g02810 | unknown protein | -1.018 | -1.248 |
| at5g35525 | unknown protein | -2.467 | -1.485 |

|  |  | **LogFC** | |
| --- | --- | --- | --- |
| **ID** | **Symbols/Description** | **AtFRh2** | **AtBG11** |
| at3g28180 | CSLC04 | -1.064 | 1.084 |
| at1g32540 | LOL1 | -1.433 | 1.055 |
| at4g13564 | MIR841A | -1.382 | 1.216 |
| at5g62280 | unknown protein | -1.333 | 1.444 |
| at5g53410 | unknown protein | -1.394 | 1.049 |
| at1g40089 | putative fructose-2,6-bisphosphatase | 1.375 | -1.039 |
| at2g36750 | UGT73C1 | 1.815 | -1.826 |
| at1g05680 | UDP-glucosyl transferase family protein | 1.793 | -1.413 |
| at2g36760 | UGT73C2 | 1.336 | -1.321 |
| at1g05530 | UGT75B2 | 1.329 | -1.282 |
| at5g42760 | unknown protein | 1.132 | -1.887 |
| at3g21890 | zinc finger (B-box type) family protein | 1.151 | -1.782 |
| at5g08070 | TCP17 | 1.083 | -1.396 |
| at1g68150 | WRKY9 | 1.338 | -1.162 |
| at4g15248 | zinc ion binding | 1.145 | -1.803 |
| at5g15500 | ankyrin repeat family protein | 2.62 | -1.08 |
| at1g04570 | membrane transporter family protein | 1.305 | -1.498 |
| at5g58770 | DEDOL-PP synthase, putative | 1.539 | -1.379 |
| at4g29770 | Target of trans acting-siR480/255 | 1.003 | -1.543 |
| at1g31300 | unknown protein | 1.568 | -1.086 |
