# Supplementary material for: Transcriptional Reprogramming of Arabidopsis thaliana Defence Pathways by the Entomopathogen Beauveria bassiana Correlates With Resistance Against a Fungal Pathogen but Not Against Insects
Source: Front Microbiol. 2019 Mar 29;10:615. doi: 10.3389/fmicb.2019.00615 (PMC6449843; doi:10.3389/fmicb.2019.00615)
Supplement: Supplementary file 6 [file Table_6.docx]

Table S6 Primer sequences

| **Genes** | **Accession number** | **Sequence 5'-3' (span ex-ex junction)** | | **Product length** |
| --- | --- | --- | --- | --- |
|  |  | **Forward** | **Reverse** |  |
|  |  |  |  |  |
| **Reference genes** |  |  |  |  |
| Actin-2 | At3G18780 | ACATTCCAGCAGATGTGGATCTC | ACCCCAGCTTTTTAAGCCTTTG | 111 |
| GAPDH | At1G13440 | TTGGTGACAACAGGTCAAGCA | AAACTTGTCGCTCAATGCAATC | 72 |
| EF1a | AT5G60390 | TGAGCACGCTCTTCTTGCTTTCA | GGTGGTGGCATCCATCTTGTTACA | 76 |
|  |  |  |  |  |
| **Target genes** |  |  |  |  |
| AXR5, | AT4G14560 | CAGAAGCAACAACAAGCGCA | CCAACGATTTGTGTTTTTGCAGG | 78 |
| ACS4 | AT2G22810 | TCTTGCGGAAAATCAGCTATGC | AACAGGCTGCGTCTGTGTTT | 71 |
| GLIP1 | AT5G40990 | CTGGCCGTATGGTCAAACAACA | CATGCGTACTCCGCGATGA | 90 |
| Chitinase | At2G43570 | TTCGGTGCTTCCATCTCCAA | TCAATGTAACACATGAACCCTGTTT | 83 |
| MYB 122 | At1G740800 | TTCCGGACAAAGCTGGACTC | TCTTGGCTAAACTCTCCACGTT | 100 |
| WRKY 63 | At1G66600 | TCATCAAAGAAGAAGATTTGCGGAG | CGTCATCAAGGCGGGGATT | 79 |
| ARR11 | AT1G67710 | GGATGCGATCACAAAGCTGG | TTCTGAAGGTGGCTTGCAACA | 92 |
