# Supplementary material for: Transcriptional Reprogramming of Arabidopsis thaliana Defence Pathways by the Entomopathogen Beauveria bassiana Correlates With Resistance Against a Fungal Pathogen but Not Against Insects
Source: Front Microbiol. 2019 Mar 29;10:615. doi: 10.3389/fmicb.2019.00615 (PMC6449843; doi:10.3389/fmicb.2019.00615)
Supplement: Supplementary file 7 [file Image_1.pdf]

Supplementary Figures

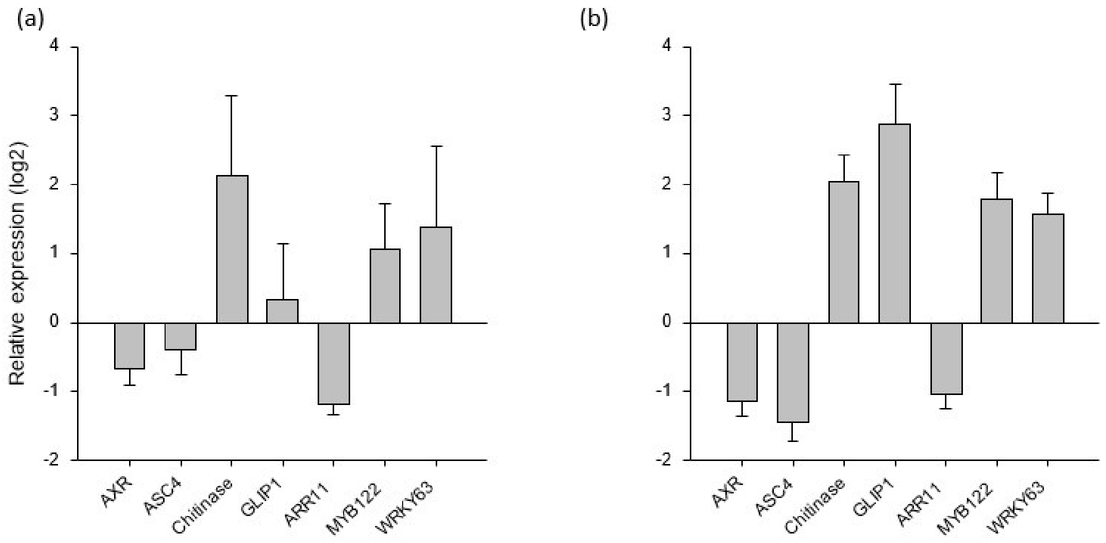

Fig. S1: Validation of microarray analysis and comparison for relative expression of *AXR*, *ACS4*, *chitinase*, *GLIP1*, *ARR1*, *MYB122* and *WRKY63* by RT-qPCR (a) and by microarray (b). RT-qPCR data presented as log_2 relative levels of gene expression calculated over three biological replicates and three reference genes using the $2^{-\Delta\Delta CT}$ method. Microarray data for expression level are calculated over four biological replicates and presented as log fold change > 1 and < -1. Error bars present standard errors of the mean.
